# The role of hypoxic microenvironment in autoimmune diseases

**DOI:** 10.3389/fimmu.2024.1435306

**Published:** 2024-11-07

**Authors:** Xun Gong, Su-Yin Yang, Zhen-Yu Wang, Min Tang

**Affiliations:** ^1^ Department of Rheumatology and Immunology, Affiliated Hospital of Jiangsu University, Zhenjiang, China; ^2^ School of Life Sciences, Jiangsu University, Zhenjiang, China

**Keywords:** hypoxic microenvironment, autoimmune diseases, rheumatoid arthritis, hypoxia-inducible factor-1, immune cells

## Abstract

The hypoxic microenvironment, characterized by significantly reduced oxygen levels within tissues, has emerged as a critical factor in the pathogenesis and progression of various autoimmune diseases (AIDs). Central to this process is the hypoxia-inducible factor-1 (HIF-1), which orchestrates a wide array of cellular responses under low oxygen conditions. This review delves into the multifaceted roles of the hypoxic microenvironment in modulating immune cell function, particularly highlighting its impact on immune activation, metabolic reprogramming, and angiogenesis. Specific focus is given to the mechanisms by which hypoxia contributes to the development and exacerbation of diseases such as rheumatoid arthritis (RA), systemic lupus erythematosus (SLE), multiple sclerosis (MS), and dermatomyositis (DM). In these conditions, the hypoxic microenvironment not only disrupts immune tolerance but also enhances inflammatory responses and promotes tissue damage. The review also discusses emerging therapeutic strategies aimed at targeting the hypoxic pathways, including the application of HIF-1α inhibitors, mTOR inhibitors, and other modulators of the hypoxic response. By providing a comprehensive overview of the interplay between hypoxia and immune dysfunction in AIDs, this review offers new perspectives on the underlying mechanisms of these diseases and highlights potential avenues for therapeutic intervention.

## 1 Introduction

The human immune system is a vital defense mechanism that comprises immune organs, cells and molecules, playing a crucial role in identifying and eliminating invading pathogens while maintaining the body’s internal environment stability and physiological balance. However, when the immune system becomes dysregulated, it can generate responses against self-antigens, leading to the attack of normal tissue cells and development of autoimmune diseases (AIDs) (1, 2). AIDs encompass a broad range of disorders triggered by factors such as the appearance of self-antigens, immune dysregulation, cross-reactivity, and genetic predisposition. Clinical AIDs mainly include rheumatoid arthritis (RA), systemic lupus erythematosus (SLE), multiple sclerosis (MS), dermatomyositis (DM) and other diseases (1–3). The onset and progression of AIDs are closely associated with this prominent hypoxic microenvironment (4, 5). Notably, hypoxia-dependent and non-hypoxia-stimulated regulatory pathways mediated by hypoxia-inducible factor-1 (HIF-1) in the hypoxic microenvironment play a pivotal regulatory role in the pathogenesis of AIDs (6, 7). The Hypoxic Microenvironment can be defined as a pathophysiological state in which the concentration of oxygen in a particular tissue area is significantly reduced (8). The formation of this microenvironment is the result of the interaction of multiple factors. As target tissues undergo destruction, a large number of immune cells, including macrophages, neutrophils, myeloid-derived suppressor cells (MDSCs), and T and B lymphocytes, infiltrate and become activated. This infiltration contributes to the formation of a hypoxic microenvironment. The ongoing inflammatory reactions and aberrant immune activation in this microenvironment lead to increased oxygen consumption, further exacerbating local tissue hypoxia (4, 5, 9). Conversely, local tissue hypoxia intensifies inflammatory reactions and immune dysfunction, further exacerbating the imbalance between oxygen supply and demand in local areas. Hypoxia, characterized by an imbalance between oxygen supply and consumption within cells, is an important feature of AIDs (10, 11). In patients with AID, under hypoxic microenvironment conditions, immune cells undergo excessive activation, abnormal blood vessel proliferation, metabolic reprogramming, and deposition of immune complexes, disrupting self-recognition, interfering with immune tolerance, and promoting the formation of autoantibodies, thus triggering immune reactions between autoantibodies or sensitized lymphocytes and self-tissue antigens (12, 13). Moreover, factors such as adaptability to hypoxic environments, drug resistance, and immune evasion are crucial determinants affecting the progression and prognosis of AIDs (14–17). Therefore, in this review, we summarize the impact of the hypoxic microenvironment on AIDs and elucidate relevant molecular mechanisms, providing guidance for further exploration of the molecular mechanisms underlying the occurrence and development of AIDs in hypoxic environments.

## 2 Overview of hypoxic microenvironment and hypoxia-inducible factors

**Figure 1:**
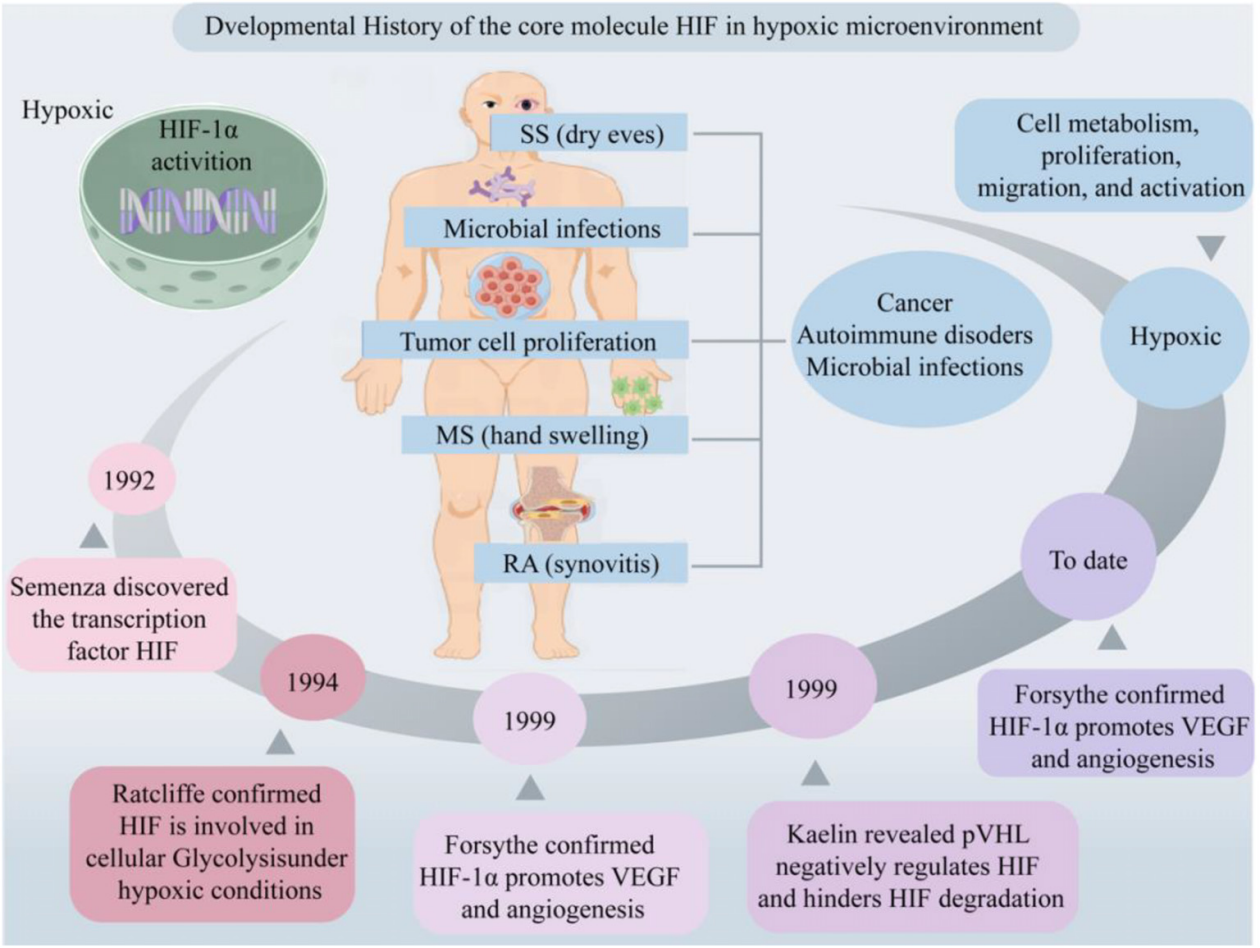
Developmental history of the core molecule HIF in hypoxic microenvironment. Hypoxic microenvironments are involved in cell metabolism, proliferation, migration and activation, mediating the occurrence of diseases such as cancer, AIDs and microbial infections. Created with figdraw.com.

In 1992, Semenza et al. discovered the transcription factor HIF associated with hypoxic stress under low-oxygen conditions (18). Subsequently, Ratcliffe et al. confirmed HIF’s involvement in cellular glycolysis, demonstrating that under hypoxic conditions, the expression levels of phosphoglycerate kinase (PGK) and lactate dehydrogenase A (LDHA) are significantly upregulated (19). Kaelin et al. elucidated that the tumor suppressor protein Von Hippel-Lindau Tumor Suppressor Protein (pVHL) negatively regulates HIF, and loss of pVHL hinders HIF degradation, thereby promoting tumorigenesis (20, 21). Forsythe et al. confirmed that the upregulation of HIF-1α facilitates the expression of vascular endothelial growth factor (VEGF), whereas the absence of HIF-1α inhibits angiogenesis, resulting in embryonic death (22, 23). In recent years, the impact of hypoxic microenvironments on HIF expression and its multifaceted involvement in cellular processes, including metabolism, proliferation, migration, and activation, has garnered considerable attention from researchers, rendering it a prominent and focal area of investigation. Furthermore, its important role in various diseases, such as cancer, autoimmune disorders, and microbial infections, underscores its significance in biomedical research (**Figure 1**).

### 2.1 Structure of HIF in hypoxic microenvironment

In hypoxic conditions, HIF serves as the primary signaling molecule, with HIF-1α exhibiting high sensitivity to low oxygen and serving as the core regulatory molecule in the body’s response to hypoxia (6). HIF is a heterodimer composed of an oxygen-sensitive α subunit and a constitutively expressed β subunit. The α subunit comprises three isoforms: HIF-1α, HIF-2α, and HIF-3α, which are regulated by oxygen. It contains an oxygen-dependent degradation domain (ODD) crucial for HIF degradation regulation (4). Under normoxic conditions (21% O_2_) HIF exhibits some expression, yet the synthesized proteins undergo rapid ubiquitination and subsequent degradation via the oxygen sensor prolyl hydroxylases (PHDs)/HIF-1α/pVHL pathway (24). Only under hypoxic conditions can HIF-1 achieve stable expression (25). Reports suggest that HIF-1α is upregulated under intense hypoxia or early hypoxia (<24 hours), while HIF-2α and HIF-3α are highly expressed under mild or chronic hypoxia (>24 hours) (26, 27). Moreover, another study found that HIF-1α is widely expressed and regulates oxygen homeostasis most significantly in mammals, whereas HIF-2α exhibits strong tissue specificity, with its functions overlapping or differing from those of HIF-1α (28). The β subunit includes HIF-1β, which can be expressed in the cytoplasm or nucleus, unaffected by oxygen concentration, playing a role in maintaining HIF conformation and stable expression (4). Collectively, this indicates that different subunits of HIF have slightly different expression patterns and functional roles in the hypoxic microenvironment.

### 2.2 Function of HIF in hypoxic microenvironment

**Figure 2:**
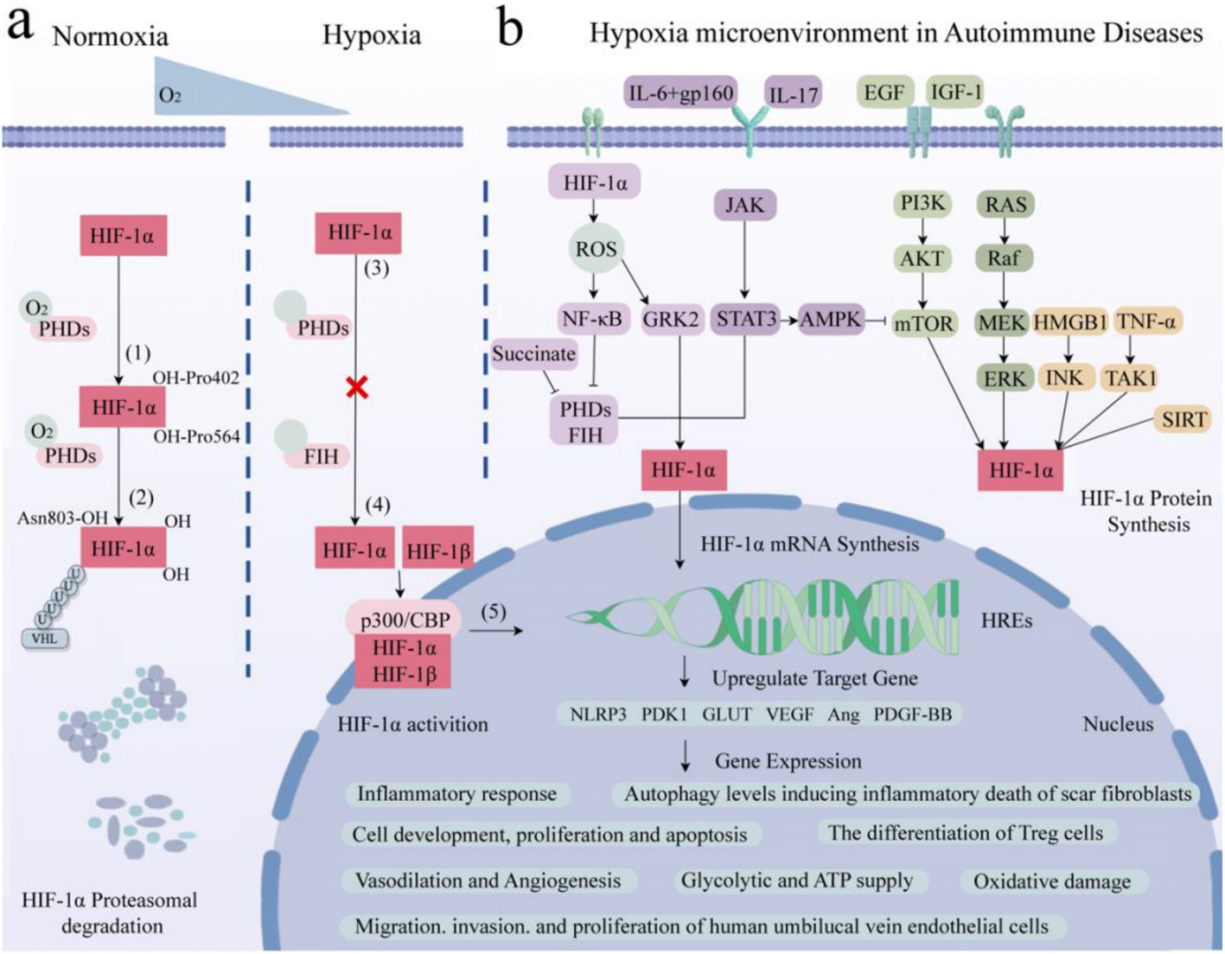
Role of HIF-1α in hypoxia-induced pathways and gene expression in AIDs. **(A)** Core molecule HIF-1α is proteasomal degradation (1). Two residues of HIF-1α, Pro402 and Pro564, were hydroxylated by PHDs under normal oxygen (2). The affinity between HIF-α and pVHL after hydroxylation is enhanced to form a stable complex. Attracts the accumulation of multiple Ub, resulting in HIF-1α degradation. (3) Hypoxia up-regulates intracellular ROS, inhibits PHDs and FIH, and HIF-1α accumulates in cytoplasm without degradation. (4) HIF-1α enters the nucleus and binds to HIF-1β and cofactor p300/CBP to form a stable heterodimer. (5) The dimer recognizes and binds to HRE and activates transcription of downstream genes. **(B)** Related signaling pathways and target gene expression in AIDs. AID patients have local hypoxic microenvironments, which regulate HIF-1α through the interaction of PHDs/HIF-1α/pVHL, NF-κB, JAK/STAT, PI3K/AKT/mTOR and MAPK signaling pathways. It is involved in inflammatory response, glucose metabolism and ATP supply, vasodilation and angiogenesis, inducing apoptosis, regulating autophagy level, promoting cell development and antioxidant damage. Created with figdraw.com.

Under normoxia conditions, the hydroxylation of Pro402 and Pro564 residues within the ODD domain of HIF-1α, catalyzed by oxygen, iron, and 2-oxoglutarate, results in conformational alterations that bolster its affinity for PHD enzyme recognition. This, in turn, initiates the subsequent association of HIF-1α with pVHL, leading to the formation of a stable HIF-1α/pVHL complex (29). This complex subsequently undergoes ubiquitination by an array of ubiquitin proteins (Ub), ultimately culminating in the proteasomal degradation of HIF-1α under normoxic conditions (30, 31) (**Figure 2A**). Under hypoxic conditions, the functionality of HIF is primarily mediated by the α subunit, where intracellular oxygen concentration affects the protein stability and transcriptional activity of HIF-1α (6). The upregulation of reactive oxygen species (ROS) expression inhibits the enzymatic activity of PHDs and factor-inhibiting HIF (FIH), subsequently resulting in the accumulation of HIF-1α within the cytoplasm (32, 33). FIH hydroxylates the transactivation domain (TAD) of HIF-1α at the C terminus, where the asparagine residue Asn803 significantly reduces the transcriptional activity of HIF-1α (34). During this process, cytoplasmic HIF-1α translocations to the nucleus, where it integrates with HIF-1β and cofactor proteins p300/CBP to form a heterodimer, thereby rendering it less vulnerable to proteasomal degradation. This heterodimer subsequently recognizes and binds to hypoxic response elements (HREs), thereby activating downstream gene transcription (4). This, in turn, promotes glucose uptake and metabolism, upregulates glycolysis via the activation of glucose transporter 1 (GLUT1) (35), stimulates erythropoiesis through the upregulation of erythropoietin (EPO) essential for red blood cell production, and mediates vascular angiogenesis via VEGF (22, 36). This illustrates that HIF activates a series of adaptive responses in hypoxic microenvironments by regulating its stability and transcriptional activity to maintain cell and tissue function and survival.

### 2.3 Activation of HIF in hypoxic microenvironment

Studies have found that the classical mitogen-activation protein kinase (MAPK), including its regulated extracellular protein kinases (ERK) subfamily, play crucial roles in the hypoxic microenvironment, activating HIF-1α, protecting cell development, and avoiding oxidative damage (37). Additionally, epidermal growth factor (EGF) and insulin-like growth factor-1 (IGF-1) activate the phosphoinositide 3-kinase (PI3K)/protein kinase B (PKB/AKT) signaling pathway, activating the mechanistic target of rapamycin kinase (mTOR). This cascade promotes HIF-1α expression and subsequently upregulates VEGF expression, ultimately promoting vasodilation (36). Furthermore, signal transducer and activator of transcription 3 (STAT3) can inhibit the mTOR/HIF-1α pathway by activating AMP-activated protein kinase (AMPK), leading to reduced cell proliferation and increased apoptosis (38). Meanwhile, Janus tyrosine kinase (JAK) can phosphorylate and activate downstream STAT3, upregulating nucleotide-binding oligomerization structure-like receptor family pyrin domain protein 3 (NLRP3) expression and thereby exacerbating inflammatory responses (39). In parallel, IL6 activates the STAT3/HIF-1α pathway through interaction with the gp160 protein, regulating the differentiation of Foxp3^+^ regulatory T cells (Treg). Conversely, IL-17 regulates autophagy levels through the STAT3/HIF-1α signaling pathway, ultimately inducing inflammatory death of scar fibroblasts (40–42).

It has been reported that tumor necrosis factor α (TNF-α) can bind with transforming growth factor-activated kinase 1 (TAK1), upregulating HIF-1α expression, thereby promoting glycolysis (43). Similarly, Hypoxia-induced high mobility group proteins 1 (HMGB1) can activate the c-Jun N-terminal kinase (JNK), which, in turn, stimulates the HIF-1α/VEGF signaling axis, facilitating angiogenesis (44). Additionally, the silence information regulator (SIRT) is a class of nicotinamide adenine dinucleotide^+^-dependent deacetylases. SIRT1 protects HIF-1α from deacetylation by binding to its inhibitory domain, while inhibiting SIRT2 downregulates fibroblast phosphorylation (45, 46). Overexpression of SIRT6 inhibits the ubiquitin proteasome system and promotes HIF-1α accumulation, upregulating the expression of VEGF, angiopoietins1 (Ang1), Ang2, and platelet-derived growth factor-BB (PDGF-BB), consequently enhancing migration, invasion, and proliferation of human umbilical vein endothelial cells (47, 48). This suggests that within the hypoxic microenvironment, the central regulatory factor HIF-1α is orchestrated by numerous signaling pathways apart from the oxygen sensor pathway PHDs/HIF-1α/pVHL, encompassing MAPK, PI3K/AKT/mTOR, and JAK/STAT among others (**Figure 2B**). Through intricate crosstalk with these diverse signaling cascades, HIF-1α modulates the secretion of inflammatory factors and participates in the regulation of various cellular processes, including survival, proliferation, metabolism, angiogenesis, and inflammatory responses.

Furthermore, in addition to the canonical activation of HIF-1α under hypoxic conditions through the aforementioned signaling pathways, this transcriptional factor can also be elicited by diverse non-hypoxic stimuli, including the accumulation of vitamin B1 (thiamine) deficiency (49), metabolic intermediates (notably succinate) (44), oxidative stress (32, 33), inflammatory responses, and metabolic reprogramming (50–52). Thiamine deficiency leads to an accumulation of metabolic intermediates, such as pyruvate and lactate, which stabilize HIF-1α through inhibition of prolyl hydroxylases (PHDs), thereby inducing HIF-1α-mediated gene expression (49). Succinate, serving as an upstream activator of HIF-1α, is translocated from the mitochondria to the cytoplasm, where it inhibits PHD activity, leading to the activation of HIF-1α. This, in turn, upregulates interleukin-1β (IL-1β) expression and triggers inflammatory responses (44, 53). Additionally, due to oxidative stress, intracellular reactive oxygen species (ROS) levels are elevated. ROS stimulate nuclear factor kappa-B (NF-κB), which downregulates PHDs and HIF, leading to cytoplasmic accumulation of HIF-1α and subsequent activation of downstream signaling molecules (32, 33). Increased ROS can induce upregulation of G protein-coupled receptor kinase 2 (GRK2)/HIF-1α and induce pyroptosis through the NLRP3 inflammasome-mediated pathway (54). Conversely, low levels of HIF-1α downregulate downstream target genes such as phosphoinositide-dependent protein kinase-1 (PDK1) and GLUT1, thereby regulating the glycolytic pathway and intracellular adenosine triphosphate (ATP) supply (35).

The Warburg effect, also known as aerobic glycolysis, denotes a significant intensification of the glycolytic pathway under aerobic conditions, characterized by augmented glucose uptake and lactate production (50–52). Under normal aerobic conditions, cells primarily depend on mitochondrial oxidative phosphorylation for energy production, with the glycolytic pathway exhibiting a relatively subordinate role. However, in the context of the Warburg effect, cells exhibit a notable enhancement of the glycolytic pathway even when oxygen is present, resulting in increased glucose consumption and lactate generation. This metabolic reprogramming is crucial for rapid cell proliferation and adaptation to hypoxic environments, frequently observed in tumors cell and inflammatory conditions. Lactate accumulation, a byproduct of enhanced glycolysis, has been shown to play a key role in immune regulation, particularly in pro-inflammatory responses. Lactate not only facilitates the metabolic reprogramming in various immune cells but also induces senescence and inflammation, promoting autoimmune pathological processes (55). The persistence of an autoimmune response can also be associated with the Warburg effect (50, 56). HIF-1α serves as a downstream effector of the mTOR signaling pathway, regulates the synthesis of diverse proteins and enzymes, modulates cellular responses to hypoxic conditions, and facilitates glycolytic metabolism (57, 58). It plays an important role in modulating the Warburg effect through the upregulation of glycolytic gene expression, particularly GLUT (35), hexokinase, and lactate dehydrogenase (LDH), which enhances cellular glucose uptake, utilization, and lactate production. Research have shown that PDGF activates the PI3K/AKT/mTOR/HIF-1α signaling pathway, thereby enhancing the Warburg effect in pulmonary artery smooth muscle cells (59). MARK kinases, members of the AMPK-related kinase family, potentiate the AMPKα1/mTOR/HIF-1α signaling axis, contributing to the Warburg effect and cell proliferation in non-small cell lung cancer (60). We hypothesize that in the hypoxic microenvironment, the activation of HIF-1α, via the mTOR signaling pathway, may further exacerbate the Warburg effect, stimulating cell proliferation and adaptation. Additionally, this augmentation may potentially exacerbate autoimmune pathological processes associated with hypoxia.

Collectively, the complex interactions between HIF-1α and its diverse hypoxia-dependent and non-hypoxia-stimulated regulatory pathways form a specific, multifaceted hypoxia-responsive pathway that is critical for cellular adaptation to changing environments and for orchestrating inflammatory responses. This understanding of HIF-1α’s versatility and complexity provides valuable insights into the development of therapeutic strategies targeting this pathway for the treatment of inflammation-related diseases.

## 3 Hypoxic microenvironment and immune cells

### 3.1 Neutrophils

Neutrophils are the core cells of innate immunity. Their overactivation results in accumulation in inflammatory areas, where they generate ROS, activate proteases of soluble proteins, induce innate and adaptive immune responses, release neutrophil extracellular traps (NETs), mediate gene expression and cell signaling, cellular metabolism, ultimately precipitating local tissue damage (61). It has been reported that neutrophils in the peripheral blood and synovial fluid of RA patients can promote ROS generation. Inflammatory cytokines secreted in the inflammatory area, such as granulocyte-macrophage colony stimulating factor (GM-CSF), TNF-α, IL-1β, and interferon, can activate HIF-1α to inhibit neutrophil apoptosis (62, 63). Hypoxia can promote the activation of NF-κB and HIF-1α in the joints of RA patients, inhibit cell apoptosis, regulate neutrophil cytoplasmic retention, and thus prolong RA inflammation (64). Additionally, research has demonstrated abnormal ROS generation in neutrophils in the peripheral blood of SLE patients, with elevated levels of O_2_, H2O2, and HO compared to normal individuals (65). This indicates the involvement of HIF-1α in neutrophil aggregation and secretion of inflammatory factors. Belimumab, approved in the UK for the treatment of SLE patients, inhibits B-lymphocyte stimulator (BLyS) primarily stored in neutrophils (66). In RA patients, disease-modifying anti-rheumatic drugs (DMARDs) targeting TNF significantly downregulate the expression of neutrophils and activate NF-κB (67). JAK inhibitors, such as Baricitinib and Tofacitinib, are small molecule drugs that hinder neutrophil migration and ROS generation by suppressing the secretion of inflammatory factors (68). This implies that hypoxic microenvironment can enhance the pro-inflammatory activity of neutrophils, increase oxidative stress and tissue damage, which may play a role in the inflammatory response of multiple AIDs. Modulating the local hypoxic microenvironment in AIDs to curb neutrophil activation and attenuate target organ damage is also one of the mechanisms by which biological agents exert their effects.

### 3.2 Macrophages

In a hypoxic environment, the activation of HIF-1α specifically promotes glycolysis in monocytes and macrophages, thereby enhancing their antigen presentation capabilities and secretion of inflammatory factors (69). Furthermore, the upregulation of HIF-1α in macrophages contributes to the clearance of infections by enhancing bactericidal effects against pathogens such as mycobacterium tuberculosis (70). Macrophages are categorized into pro-inflammatory M1-type and anti-inflammatory M2-type, both of which participate in the activation of HIF-1α under certain conditions (71, 72). Research has demonstrated that HIF-1α can promote the expression of NF-κB in macrophages induced by lipopolysaccharide (LPS) (73). Following the activation of pro-inflammatory M1 macrophages, the activation of NF-κB, upregulation of HIF-1α, and the presence of ROS synergistically contribute to the polarization of macrophages towards the M1 phenotype (74). Blocking the NF-κB signaling pathway in HIF-1α promotes the transition of macrophages from M1 to M2 phenotype, which is crucial for the treatment of RA (75). In addition, Jing revealed that immunoglobulin G (IgG) stimulation of human and murine macrophages results in the production of numerous proinflammatory mediators, including IL-1β, which is dependent on HIF-1α and mTOR signaling, leading to metabolic reprogramming and subsequently driving inflammation in AIDs, such as RA, SLE, SSs, Sjögren’s syndrome (SS) and vasculitis (76). Collectively, these findings suggest that inhibiting metabolic reprogramming of macrophages and downregulating HIF-1α expression in the hypoxic microenvironment may represent viable therapeutic strategies for alleviating tissue inflammation in patients with AIDs.

### 3.3 Dendritic cells

Research has found that in the hypoxic microenvironment, HIF-1α can directly bind to long non-coding ribonucleic acid Dpf3 (LncRNA Dpf3) or p38 MAPK, subsequently inhibiting the glycolytic metabolism and migratory potential of dendritic cells (77). Concurrently, the interaction of HIF-1α with the PI3K/AKT pathway enhances the migratory abilities of dendritic cells (78), revealing its dual roles in different signaling pathways. Additionally, downregulation of HIF-1α can inhibit its antifungal immune response (79), further emphasizing its critical role in immune defense. Some findings reveal that the absence of HIF-1α impairs the ability of dendritic cells to induce regulatory Treg, resulting in exacerbated intestinal inflammation in mice (80). Additionally, the downregulation of HIF-1α markedly diminishes the level of granzyme B released by DCs during T cell activation (81), highlighting the indispensable role of HIF-1α in facilitating DC-mediated T cell activation. Other studies have also demonstrated that HIF-1α exerts a suppressive effect on IL-12 production in dendritic cells, thereby restricting the development of Th1 cells and subsequently influencing the balance of immune responses (82).Collectively, these findings suggest that the hypoxic microenvironment, by modulating the expression of HIF-1α, may regulate dendritic cell migration capability, glucose metabolism, immune defense capabilities, and T cell activation, thereby exerting profound effects on disease progression.

### 3.4 Natural killer cells

Furthermore, research has revealed that under hypoxic conditions, IL-15 activates the STAT3 signaling pathway, while IL-2 stimulates the PI3K/mTOR signaling pathway, thereby maintaining HIF-1α stability and enhancing the innate immune defenses of natural killer cells against microbial infections and cancer (83, 84). Moreover, during cytomegalovirus infection, downregulation of HIF-1α expression has been shown to impact the survival of natural killer cells (85). This indicates that regulating natural killer cells within the hypoxic microenvironment may be involved in anti-tumor and antiviral processes.

### 3.5 B cells

In the hypoxic microenvironment, HIF-1α can regulate B cell development, differentiation, maturation, and antibody secretion (86). It has been shown that oxygen level plays a critical role in regulating B cell differentiation, where hypoxia promotes differentiation into unique CD27++ B cells with enhanced antibody secretion capacity upon restimulation (87).HIF-1α is a key regulator of B cell glycolytic metabolism in the germinal center, and low expression of HIF-1α inhibits B cell responses, leading to abnormal class switching (88). Additionally, HIF-1α serves as a critical transcription factor for B cells producing IL10 in AIDs. Downregulation of HIF-1α significantly reduces the number of IL-10 secreting B cells, exacerbating collagen-induced arthritis and experimental autoimmune encephalomyelitis (EAE) (89). It has been speculated that the use of HIF activators in clinical practice may improve the local hypoxic microenvironment and regulate B cell-related AIDs.

### 3.6 T cells

**Figure 3:**
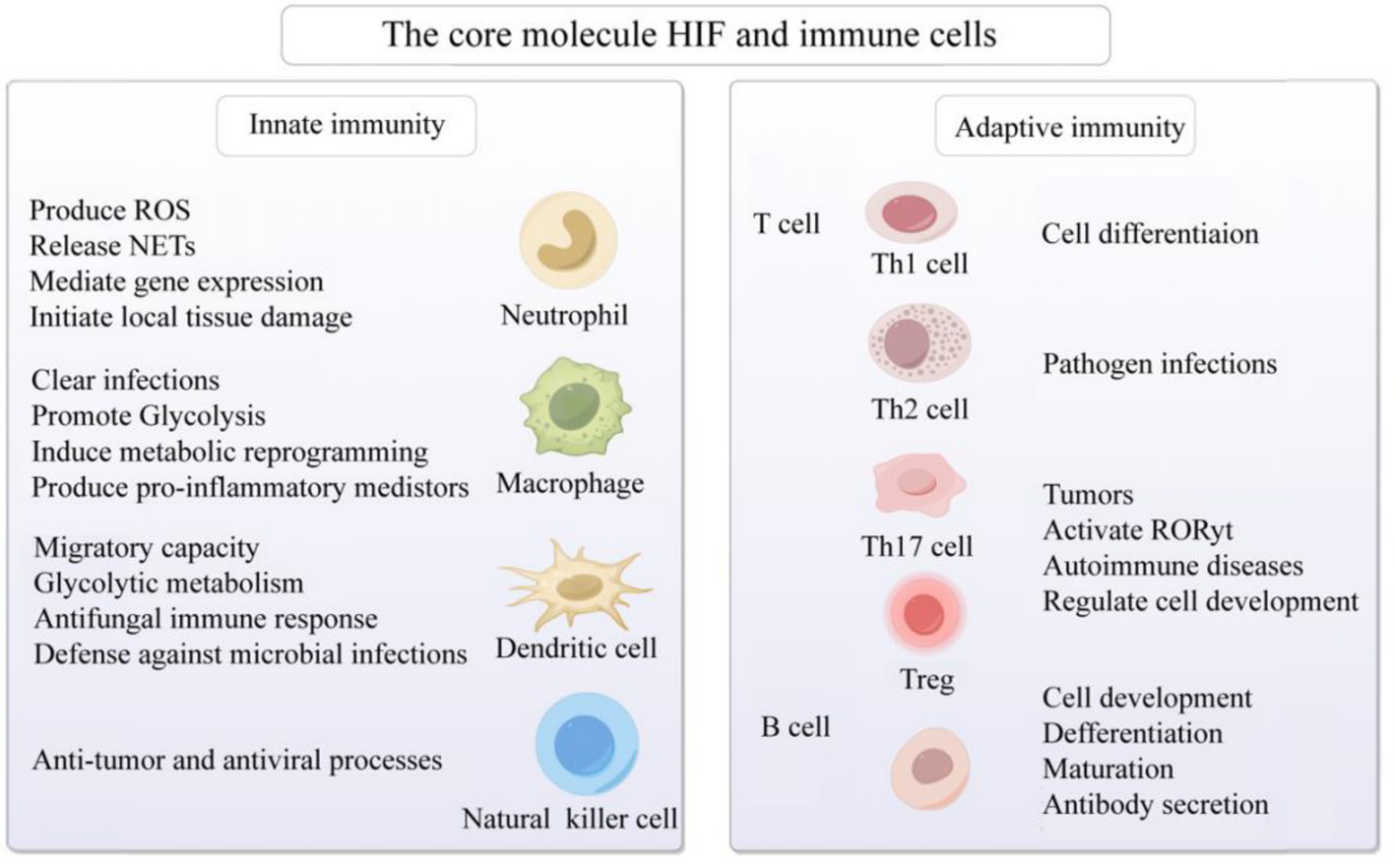
Activation of the core molecule HIF and immune cells in hypoxic microenvironment. The core molecule HIF in hypoxic microenvironment affects neutrophils, macrophages, dendritic cells, natural killer cells, B cells and T cells, mediates congenital and adaptive immune responses, and participates in cell development, differentiation and maturation, gene expression, metabolic reprogramming, anti-tumor, prevention of viral infection, and AIDs. Created with figdraw.com.

Under hypoxic conditions, the T cell receptor (TCR) engages with HIF-1α, thereby activating HIF-responsive genes. This interaction modulates the equilibrium among Th1, Th2, Th17, Treg, and other T cell subsets, subsequently regulating inflammatory responses (90–92). Investigations have revealed that, under hypoxic conditions, in antigen-presenting cells lacking HIF-1α, overexpression of STAT3 significantly inhibits Th1 proliferation (93). Conversely, micro-ribonucleic acid (microRNA) 182, an inhibitor of HIF-1α, expedites Th1 cell differentiation, potentially becoming a novel therapeutic target in the progression of autoimmune inflammation (94). During pathogen infection, HIF-1α expression is upregulated in Th2 cells, thereby fostering their proliferation (95). Downregulation of HIF-1α inhibits Th0 differentiation into Th17 cells and reduces the expression of the Th17 transcription factor RAR-related orphan receptor gamma t (RORγt) (96). HIF-1α regulates the development of Treg and Th17 cells by directly transcribing and activating RORγt expression and inhibiting FoxP3 transcriptional activity (97). Furthermore, research has found that HIF-1α promotes the development of Th17 cells while inhibiting the differentiation of Treg cells (98). HIF-1α upregulates the expression of CD73 in Treg cells, enhancing their suppressive capabilities through CD73 binding and facilitating a metabolic shift towards aerobic glycolysis (99). Elevation of O2 levels at tumor sites downregulates HIF-1α, affecting the metabolism of tumor cells and Treg cells, and ultimately reducing cancer cell proliferation (100). Additionally, in SLE patients’ T cells, SIRT2 promotes Th17 cell differentiation and inhibits CD4^+^ T cell production of IL-2, correcting abnormal expression of IL-17A and IL-2, and benefiting CD4^+^ T cells in lupus prone mice and SLE patients (101). It is speculated that targeting HIF-1α to regulate the differentiation, metabolism, and function of T cells in a low oxygen microenvironment may have potential therapeutic value for immune responses, inflammation, tumors, and AIDs (**Figure 3**).

### 3.7 Myeloid-derived suppressor cells

MDSCs, initially identified within the tumor microenvironment, represent a heterogeneous population of immature myeloid cells, comprising diverse stages of myeloid differentiation, such as myeloid progenitor cells, immature macrophages, monocytes, and dendritic cells. MDSCs exert their immunosuppressive effects by inhibiting the function of T cells, B cells, natural killer cells, and other myeloid lineages, thereby dampening immune responses and facilitating tumor progression through the suppression of antitumor immune responses (102, 103). Under normal physiological conditions, myeloid progenitor cells undergo ordered differentiation into mature granulocytes, macrophages, and dendritic cells, which are then distributed throughout various tissues and organs to execute and maintain normal immune functions. However, under pathological conditions such as tumor development, bone marrow transplantation, severe trauma, infection, inflammatory immune responses, and various AIDs, the expression of immunosuppressive factors is significantly upregulated, inhibiting the normal differentiation of myeloid progenitor cells and leading to abnormal expansion and accumulation of MDSCs. This, in turn, disrupts the balance of the immune system and exacerbates the persistence and progression of pathological states (104–108).

Recent studies have elucidated that the immunosuppressive function of MDSCs is intricately regulated by a myriad of complex metabolic pathways, with hypoxia playing a pivotal role in this regulation (109–111). HIF-1α orchestrates metabolic reprogramming in MDSCs, particularly by augmenting their glycolytic capacity, a phenomenon known as the Warburg effect. This metabolic shift enables MDSCs to sustainably generate energy in hypoxic environments, while concurrently mitigating ROS production and subsequent apoptosis, thereby safeguarding their survival and functionality (110, 112).Chiu et al. have revealed that in MDSCs derived from hepatocellular carcinoma patients, hypoxia activates HIF-1, leading to the upregulation of ectonucleoside triphosphate diphosphohydrolase 2 (ENTPD2) expression, which subsequently facilitates the enzymatic hydrolysis of extracellular ATP into 5’-AMP. This metabolic alteration, in turn, impedes the normal differentiation of MDSCs and fosters their accumulation within the tumor microenvironment (113). This underscores the significance of MDSCs as a crucial immunosuppressive cell population, whose accumulation and activation within the tumor microenvironment constitute a vital component of tumor immune evasion mechanisms. We hypothesize that inhibiting the activity of ENTPD2 under hypoxic conditions may effectively disrupt MDSC function, thereby restoring T-cell activity and function, with the potential to enhance the overall efficacy of immunotherapy.

Furthermore, HIF-1α exerts profound effects on the recruitment, activation, and immunosuppressive functions of MDSCs. Li et al. have demonstrated that TGF-β enhances the immunosuppressive capabilities of MDSCs in non-small cell lung cancer patients by activating the mTOR/HIF-1 signaling pathway, which in turn upregulates the expression of CD39 and CD73 on MDSCs (114).We postulate that the development of small molecule inhibitors or antibodies specifically targeting HIF-1α activity, or the utilization of existing mTOR inhibitors (such as rapamycin and its derivatives), may effectively impede the activation of HIF-1α by TGF-β, thereby inhibiting the recruitment and activation of MDSCs. This strategy offers novel insights and potential therapeutic avenues for alleviating immunosuppression and controlling tumor progression. Ding et al. have devised hypoxia-responsive sono-activatable semiconducting polymer nanopartners (SPNTi), which are capable of generating singlet oxygen (^1^O_2_) during sonodynamic therapy (SDT), thereby disrupting the surface shell to release hypoxia-responsive drugs (tirapazamine (TPZ) conjugates) and MDSC-targeting drug (ibrutinib) (115). In the severely hypoxic tumor microenvironment, the TPZ conjugates are activated to augment immunogenic cell death (ICD), while ibrutinib effectively mitigates the immunosuppressive effects of MDSCs. The synergistic effects of these two agents significantly potentiate antitumor immune responses (115). This innovative strategy overcomes the limitations posed by the hypoxic environment post-treatment, enabling precise targeting of MDSCs and presenting a novel, high-precision, and safe sonodynamic immunotherapy approach for cancer treatment.

Studies have demonstrated that MDSCs accumulate prominently in secondary lymphoid organs of AID patients, particularly at sites of disease manifestations such as RA, MS, and SLE (116–118). This revelation underscores the pivotal role of MDSCs in AID progression and provides a novel perspective for considering them as potential therapeutic targets. In both AID patients and corresponding animal models, the expansion and accumulation of MDSCs protect host tissues from damage caused by excessive T cell responses by inhibiting T cell activation and immune reactions (102, 119), This further indicates that MDSCs may exert a detrimental impact on immune responses by modulating the functions of CD4^+^ and CD8^+^ T cells, thereby contributing to the pathological processes of AIDs. Consequently, the development of targeted therapies aimed at precisely inhibiting the immunosuppressive functions of MDSCs at specific time points and tissue locations holds promise as a novel and critical strategy for restoring effective immune responses and promoting disease remission (118–121). In summary, hypoxia plays a pivotal role in the metabolic reprogramming, recruitment, activation, and immunosuppressive functions of MDSCs through HIF-1α. We speculate that inhibiting HIF-1α or key enzymes in the glycolytic pathway may attenuate the immunosuppressive functions of MDSCs, thereby altering the balance of immune responses and potentially contributing to the immune evasion mechanisms of AIDs.

## 4 Hypoxic microenvironment in autoimmune diseases

### 4.1 Rheumatoid arthritis

RA is a chronic and systemic AID, exhibiting a global prevalence ranging from 0.5% to 1.0%. The prevalence rate in mainland China is 0.42%, and it is hypothesized that about 5 million people are affected, with a male-to-female ratio of about 1:4. Its main pathological features encompass abnormal synovial inflammation proliferation, angiogenesis, pannus formation, as well as irreversible damage to cartilage and bones, ultimately resulting in joint deformity and disability (101, 122, 123). The pathogenesis of RA remains exceedingly intricate and is yet to be fully unraveled. In recent years, the importance of hypoxic microenvironment in the pathogenesis of RA has gradually attracted attention. The hypoxic microenvironment is the initiating factor for abnormal synovial proliferation, and it is involved in the important processes of RA occurrence and development (122–125). The synovium of RA patients frequently exhibits a hypoxic state, primarily attributable to the excessive proliferation of synovial tissue coupled with inflammatory reactions. The formation of new blood vessels is often insufficient to provide adequate oxygen to the synovium, leading to a decrease in local microenvironmental oxygen tension and the formation of a hypoxic synovial microenvironment, which in turn affects angiogenesis, inflammatory responses, matrix degradation, cartilage erosion, energy metabolism disorders, and oxidative stress (126, 127). This underscores the notion that local tissue hypoxia can exacerbate inflammation and immune dysfunction, further aggravating tissue hypoxia, thereby perpetuating a vicious cycle. Ahn et al. reported strong expression of HIF-1α in the intimal layer of macrophage-like synoviocytes (MLS) in human RA synovium (128), while HIF-2α is mainly expressed in fibroblast-like synoviocytes (FLS) in human RA synovium and collagen-induced arthritis model (CIA) (129). Related research found that compared to osteoarthritis (OA) patients, the expression of HIF in FLS and MLS of RA patients increased (128, 130). Moreover, HIF expression has been observed in chondrocytes and osteoclasts, indicating its ubiquitous presence in synovial tissues (129). Collectively, these findings suggest that distinct HIF subtypes are expressed in various synovial cell types, all of which participate in the occurrence and progression of RA.

Research has reported that the activation of PI3K/AKT/HIF-1α or NF-κB/HIF-1α pathways can stimulate the migration and invasion of FLS in RA patients (131, 132). The STAT3/HIF-1α/fascin-1 axis plays an important role in the hypoxic microenvironment of RA synovium, facilitating endothelial-to-mesenchymal transition (EndoMT) of FLS and thereby promoting migration and invasion (133). Under hypoxic microenvironment, HIF-1α regulates the migration and invasion of RA FLS and angiogenesis through the Notch-1 pathway (134). Furthermore, chemokine (C-X-C motif) ligand 12 (CXCL12) functions as a potent chemotactic and angiogenic factor, playing a role in angiogenesis. Hypoxia at the site of inflammation stimulates FLS to produce messenger RNAs such as CXCL12 and VEGF, resulting in a 50% and 132% increase in the protein levels of VEGF and CXCL12, respectively, after 24 hours of hypoxia (135). VEGF, the primary angiogenic target of HIF-1α, exhibits oxygen-dependent expression. The downregulation of HIF-1α notably diminishes VEGF-mediated angiogenesis in FLS. The activation of the PI3K/AKT/mTOR signaling pathway in the adjuvant arthritis model may be involved in inducing newly formed synovial vessels (136). Collectively, these findings suggest that HIF-1α is intricately involved in the proliferation, migration, and invasion of FLS in RA patients, alongside regulating angiogenesis and inflammatory responses.

As the disease progresses, joint erosion and bone degradation gradually intensify, representing another major characteristic of RA. The elevated expression of HIF-1α has been correlated with augmented bone erosion in RA patients (137). Knowles et al. have demonstrated that HIF-1α promotes bone resorption mediated by osteoclasts (OCs), while angiopoietin-like 4 (ANGPTL4) compensates for HIF-1α’s inadequate stimulation of OC activity and promotes the proliferation and differentiation of osteoblasts (OBs) (138). Swales et al. demonstrated that ANGPTL4 is highly expressed in OCs of RA patients in a HIF-1α-dependent fashion, contributing to bone resorption. In RA, HIF-1α induces the upregulation of receptor activator of NF-κB ligand (RANKL) in OBs, ultimately facilitating OCs-mediated bone resorption (137, 139). HIF-1α regulates the expression of Notch-1, thereby stimulating the production of matrix metalloproteinase 2 (MMP2) and MMP9 in endothelial cells (132). The interaction between HIF-1α and NF-κB promotes the enzymatic activity of MMP2 and MMP9, leading to the disruption of tissue barriers and bone materials (130). IL-38 has the potential to suppress inflammation and angiogenesis in CIA rats, upregulating osteogenic factors through the SIRT1/HIF-1α signaling pathway, thus mitigating joint damage (140). Collectively, these findings underscore the important role of HIF-1α in mediating bone destruction in FLS of RA patients.

Li et al. reported that the combination of dexamethasone (DEX) and artesunate (ART) can be used to treat RA by regulating the HIF-1α/NF-κB signaling pathway, controlling ROS clearance, and reversing macrophage polarization (75). Meanwhile, previous research findings indicate that berberine inhibits the proliferation and adhesion of FLS in RA patients, accomplishing this through modulation of the MAPK/FOXO/HIF-1α signaling pathway (141). Furthermore, Jia et al. discovered that administering roburic acid (RBA), a component of the anti-RA herb Gentiana, to RA joints can effectively block the ERK/HIF-1α/GLUT1 pathway, fostering the transition from M1 to M2 macrophage phenotype, suppressing inflammatory cytokines, and enhancing tissue repair, thereby yielding potent therapeutic outcomes for RA (142). Furthermore, Pang et al. recently found that the expression of receptor-interacting protein kinase 3 (RIPK3) is significantly increased in intestinal epithelial cells (IECs) of RA patients. Their findings reveal that HIF-1α can induce a switch from physiological cell apoptosis to pathological necrotic apoptosis by transcriptional inhibition of RIPK3, thereby initiating intestinal mucosal inflammation and exacerbating arthritis. Utilizing HIF-1α inhibitors, such as Roxadustat, has been shown to block IEC death, maintain intestinal barrier function, and ameliorate arthritic symptoms (143, 144). This further indicates that modulating HIF activity or inhibiting the inflammatory response of FLS in RA patients under hypoxic conditions may provide new insights for RA patient treatment strategies. This underscores the potential for modulating HIF activity or inhibiting the inflammatory response of FLS in RA patients under hypoxic conditions, which may potentially yield novel therapeutic avenues for RA treatment strategies.

**Figure 4:**
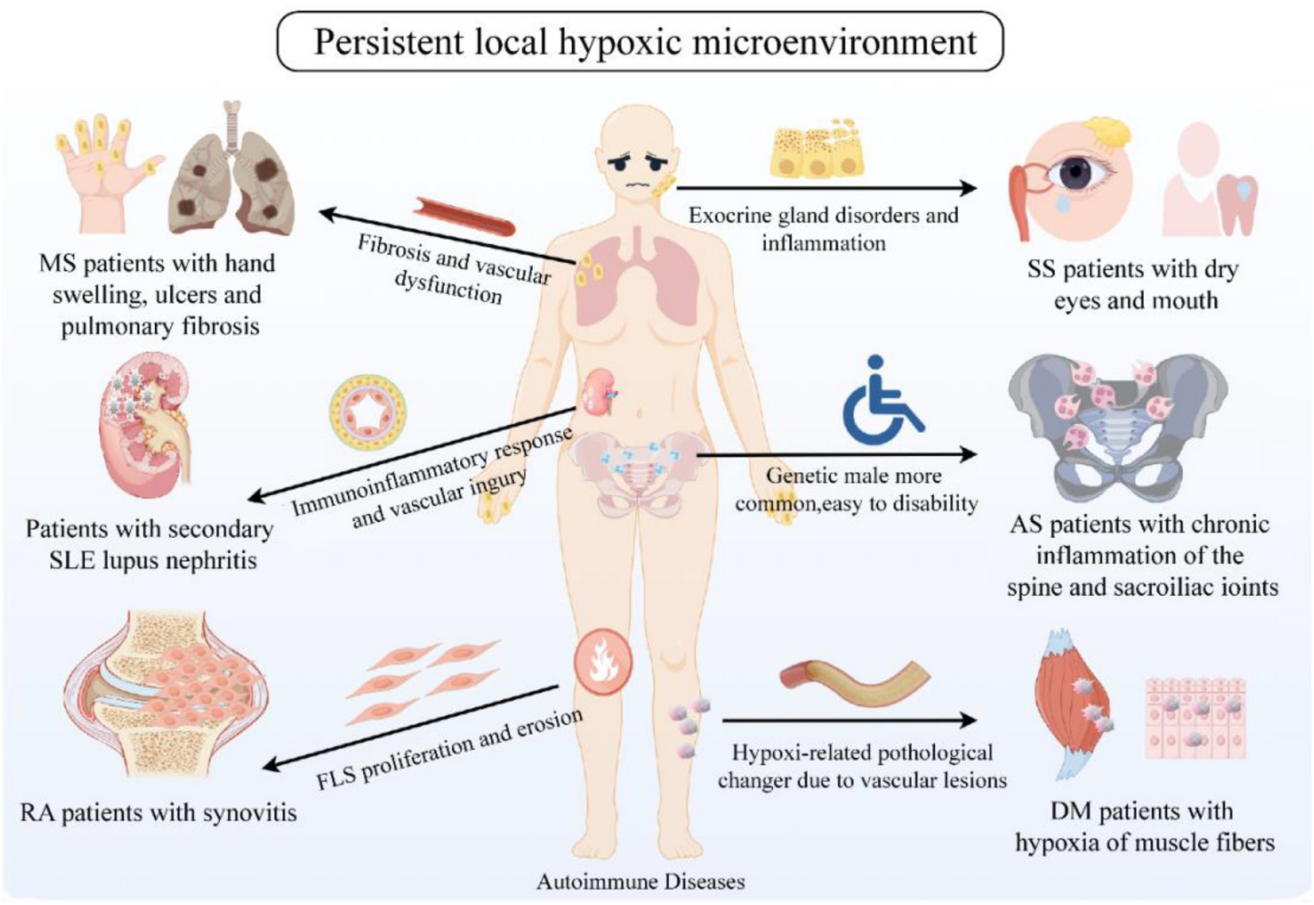
Persistent local hypoxic microenvironment in clinically common AID patients. Clinically common AIDs include RA, SLE, MS, DM, SS, AS, etc. The common feature is the continuous hypoxic microenvironment of patients. Hypoxia affects the proliferation and erosion of tissue cells, inflammatory response, vascular damage, glandular dysfunction, skin and bone fibers, etc., thus participating in the pathogenesis of AIDs. Created with figdraw.com.

Increasing evidence suggests that cells derived from the mesenchyme, especially FLS, are crucial cellular elements in RA and promising targets for future arthritis treatments. They play a critical role in mediating direct tissue damage and the continuation of complex disease processes in autoimmune joint diseases like RA. It is speculated that targeting the regulation of HIF-1α expression potentially modulate the behavior of FLS within local hypoxic microenvironments, thereby exerting a positive influence on inflammation, angiogenesis, and bone destruction in RA patients. Consequently, strategies aimed at modulating HIF-1α to ameliorate local hypoxic conditions may hold significant therapeutic potential for patients with AIDs (**Figure 4**).

### 4.2 Systemic lupus erythematosus

SLE, a prototypical AID, is characterized by recurrent autoantibody production, complement system activation, and immune complex deposition across multiple organ systems, notably lupus nephritis (LN) (145–147). Recent research has increasingly illuminated a close association between the hypoxic state in SLE patients and its occurrence and development (148). From a pathological perspective, SLE often exhibits a hypoxic state in local tissues due to abnormal activation of T and B cells, sustained (148). Moreover, metabolic reprogramming in immune cells, particularly mitochondrial dysfunction, has been linked to enhanced immune responses and tissue damage in SLE, further highlighting the role of hypoxia in driving autoimmune pathology (149). Pathologically, SLE frequently exhibits a hypoxic state in local tissues due to abnormal activation of T and B cells, sustained immune-inflammatory reactions, and vascular damage. This hypoxic state modulates SLE pathogenesis of SLE through mechanisms that include altering immune cell metabolism, enhancing inflammatory mediator production, and regulating angiogenic factor expression. Caielli et al. observed that HIF degradation defects during erythrocyte maturation in SLE patients result in the accumulation of mitochondria-dense red blood cells and subsequent mitochondrial dysfunction, contributing significantly to SLE pathogenesis (150). Another investigation revealed that in photosensitive SLE patients, circulating miR-210 and HIF-1α levels are significantly elevated and positively correlated (*r*=0.886, *P*<0.05), and miR-210, a critical hypoxia-related molecule, can regulate hypoxic signaling within hypoxic microenvironments (151, 152). Collectively, these findings underscore a close association between the hypoxic microenvironment and the initiation, progression, and severity of SLE.

Recent studies have revealed that mitochondrial activity not only influences cellular energy metabolism but also affects epigenetic modifications, such as histone methylation and acetylation, which are crucial in the regulation of immune responses under hypoxic conditions. This interaction may contribute to long-range effects on autoimmune disease progression by altering gene expression and immune cell function (153). Research has demonstrated that CD4^+^ T cell proliferation is apparent in both the peripheral blood of SLE patients and the renal tubulointerstitial compartment of individuals with proliferative lupus nephritis (PLN). These cells are capable of producing high levels of mitochondrial ROS (mtROS) in a process known as reverse electron transfer (RET). A key feature of the RET process is the accumulation of succinic acid. CD4^+^ T cells produce and secrete higher levels of succinic acid, upregulating HIF-1α, which further affects their metabolism and function, including promoting inflammation and immune response (44, 75, 101, 154). This suggests that CD4^+^ T cells play an important role in AIDs such as SLE through succinic acid accumulation, HIF-1α activation and mtROS production. We speculate that interventions targeting the activation pathway of CD4^+^ T cell subpopulations, such as down-regulation of HIF-1α or inhibition of hypoxic microenvironment, may reduce the expansion and function of CD4^+^ T cells, thereby inhibiting the initiation or persistence of SLE. In T cells of SLE patients, mTOR activation triggers ROS production, while SIRT2 concurrently upregulates IL-17A expression in these cells by modulating the mTOR complex 1 (mTORC1)/HIF-1α/RORγt signaling pathway, thereby contributing to the pathogenesis of SLE (101, 155). We speculate that downregulation of HIF-1α via the mTOR1 pathway or alleviation of the hypoxic microenvironment may attenuate the Warburg effect, impede T cell differentiation, inhibit glycolytic metabolism, and modulate inflammatory and immune responses in AID patients such as SLE.

In the hypoxic microenvironment, AKT and mTOR are located upstream of HIF-1α (36). Kshirsagar et al. discovered that downregulation of mTOR in LN patients leads to a reduction in STAT3 and Th17 cells within effector T cells, while inhibiting the AKT signaling pathway hinders the migration of Th17 cells towards inflammatory sites, effectively mitigating the chronic inflammatory process in LN (38, 156). The activation of mTORC1 precedes the onset of SLE and its associated complications, including antiphospholipid syndrome, potentially serving as an early diagnostic biomarker for the disease. The targeting of mTORC1 with mTOR inhibitors to counteract its overactivation may replace drugs with significant side effects such as prednisone or cyclophosphamide (151). It has been reported that in renal tissue macrophages, the induction of glycolysis through the mTOR/HIF-1α signaling pathway exerts an inhibitory effect on inflammation and the recruitment of neutrophils mediated by immune complexes, underscoring its important role in diseases such as LN (76). Additionally, luteolin has been shown to inhibit the expression of HIF-1α and oxidative stress in macrophages, thereby mitigating kidney damage attributed to macrophage infiltration in LN (157). Chen et al. pointed out that renal hypoxia contributes to heightened T cell infiltration and kidney damage, partially mediated by HIF-1α-induced metabolic reprogramming in lupus-prone mice (MRL/lpr mice). Targeting HIF-1α may represent a viable therapeutic approach for mitigating kidney damage in autoimmune disorders, including AIDs (158). Collectively, these findings suggest that modulating key factors within the HIF-1α signaling pathway, both upstream and downstream, can effectively optimize effector cell metabolism, diminish the proliferation and overactivation of abnormal, self-reactive cells, and subsequently ameliorate tissue inflammation in SLE patients, particularly those with LN.

Current therapeutic approaches for SLE primarily focus on the suppression of the overall immune response, utilizing medications such as corticosteroids, hydroxychloroquine, and azathioprine (158). Nonetheless, the prolonged use of corticosteroids and other forms of immunosuppression frequently results in significant adverse effects, notably infections, which have emerged as a leading cause of mortality in SLE patients (159). Targeting tissue-infiltrating immune cells has been shown to be efficacious in mitigating organ damage (160). Research has demonstrated that the utilization of HIF-1α inhibitors (PX-478) in LN mouse models effectively delays the recruitment of T cells to the kidneys, reverses the detrimental effects of T cells on tissue, and ameliorates kidney damage (158). We speculate that inhibiting HIF-1α to modulate the hypoxic microenvironment may offer novel insights into the development of therapeutic strategies for SLE.

### 4.3 Multiple sclerosis

MS is a systemic AID of unknown etiology, characterized by immune dysregulation, skin and visceral organ fibrosis, and vascular dysfunction, which are thought to be related to the pathogenesis of MS (161–163). Hypoxia is an important factor in triggering fibrosis, inducing the deposition of extracellular matrix (ECM) and increasing VEGF expression. It promotes tissue fibrosis by binding to PDGF receptors, while excessive ECM deposition exacerbates vascular lesions and hypoxia, further aggravating fibrosis (162, 164, 165). Ottria et al. found that hypoxia can drive the production of CXCL4 in MS patients’ plasmacytoid dendritic cells by stabilizing HIF-2α through excessive production of mtROS (*P*=0.0079) (166). This finding underscores the potential therapeutic value of inhibiting the mtROS/HIF-2α signaling pathway and ameliorating the hypoxic microenvironment to attenuate the detrimental effects of CXCL4 in MS and other CXCL4-driven inflammatory disorders. Additionally, Liu et al. found that hypoxia promotes MS fibroblast activity through autophagy acceleration, while autophagy-mediated hypoxia-induced 2-methoxyestradiol inhibits MS collagen synthesis and EndoMT (167). Abnormal expression and activity of HIF-1α have also been implicated in the development of various fibrotic diseases under hypoxic microenvironment conditions, including renal and cardiac fibrosis (168). Specifically, HIF-1α in human coronary artery endothelial cells has been shown to induce EndoMT and promote cardiac fibrosis through snail activation (169). Similarly, HIF-1α-mediated EndoMT leads to pulmonary fibrosis (170). Li et al. further confirmed that hypoxia participates in the pathogenesis of MS by modulating EndoMT-related genes, potentially involving transforming growth factor-β (TGF-β), NF-κB, TNF, and mTOR signaling pathways (171). Collectively, these findings emphasize the role of the hypoxic microenvironment in MS-associated fibrosis, suggesting that its regulation may represent a potential new therapeutic strategy for MS and other fibrotic diseases.

At the early stage of MS pathogenesis, vascular endothelial damage and apoptosis upregulate vasoactive substances, prompting the migration of immune cells towards the vasculature and peripheral tissues, leading to inflammation, oxidative stress, and tissue hypoxia (162, 172, 173). In turn, this inflammatory milieu, characterized by the production of inflammatory and pro-fibrotic cytokines, further exacerbates microvascular endothelial cell injury and apoptosis (174, 175), creating a vicious cycle that culminates in thrombosis, inflammation, and a myriad of clinical manifestations ranging from skin to parenchymal organs (174, 176). These observations underscore the involvement of the hypoxic microenvironment in MS-associated vascular pathology, implying that strategies aimed at improving vascular oxygenation, mitigating immunoinflammatory damage, downregulating vasoactive substances, and reversing vascular abnormalities may reduce ischemic damage to the skin and visceral organs.

Moreover, approximately 20%-40% of MS patients clinically develop subepidermal calcium salt deposits, known as cutaneous calciphylaxis (161, 177, 178). Hypoxia has been posited as a possible link between vascular dysfunction and calciphylaxis in MS (179), where vascular dysfunction with defective angiogenesis leads to recurrent episodes of ischemia-reperfusion injury, increasing the products of oxidative stress, which results in tissue hypoxia (180). Hypoxia accompanied by damage to collagen, elastin or subcutaneous fat further exacerbates tissue necrosis, releasing denatured proteins and leading to calcification (181). Compared to healthy individuals, MS patients, both with and without calciphylaxis, exhibit elevated basal expression levels of hypoxia-related proteins (GLUT1 and VEGF), with the highest levels observed in those with calciphylaxis (182). Vascular variations may predispose MS patients to finger ischemia and hypoxia-induced limb osteolysis, thereby facilitating the deposition of calcium plaques in the skin tissues (178). These observations further suggests that the hypoxic microenvironment is closely associated with the development of cutaneous calciphylaxis in MS patients and is involved in their vascular pathology and calcium salt deposition.

Given the association between hypoxia and MS pathogenesis, it is tempting to speculate that regulating HIF-1α may be possible to improve the local hypoxic microenvironment in MS patients, reducing vascular inflammation and fibrosis, and ultimately alleviating clinical symptoms.

### 4.4 Dermatomyositis

DM, an inflammatory myopathy that primarily affects skin and muscle tissues, is prevalent among young individuals and adults. Changes related to hypoxia induced by vascular alterations may be a significant pathogenic mechanism (183, 184). Recent studies have demonstrated that in DM muscle fibers, endothelial cells prominently express HIF-1α and HIF-1β, while the muscle fibers themselves exhibit expression of HIF-2α, as well as VEGF, and VEGF receptors (185). VEGF is involved in hypoxia regeneration and angiogenesis, and the upregulation of hypoxia-related proteins may be intimately associated with the reduced adaptation mechanisms of muscle tissues to decreased blood supply in DM patients. This suggests that HIF-1α may promote angiogenesis in response to hypoxia by up-regulating the expression of genes such as VEGF. Nonetheless, in the context of DM, the aforementioned adaptive mechanism may be compromised, resulting in inadequate blood supply to muscle tissues and subsequent exacerbation of muscle damage. In addition, Preuße et al. found that HIF-1α was positive in muscle biopsy samples of juvenile DM (jDM, DM patients under 16 years old), accompanied by significantly elevated HIF-1α expression levels as indicated by mRNA data. Using distinct HIF-1α antibodies for staining macrophages, dual-positive immunofluorescence for HIF-1α^+^ and CD206^+^ macrophages was detected in proximity to capillaries (186), indicating that vascular neogenesis in jDM patients is regulated by the VEGF/HIF pathway, inducing HIF-1α and promoting CD206 expression in macrophages, which may lead to hypoxic damage to capillaries in DM patients. Luna et al. reported the presence of HIF-1α within the muscle fibers of DM patients, the firefly/kidney assay suggested that retinoic acid-inducible gene I (RIG-I), a newly described HIF-induced gene containing HRE, was essential for the transcriptional response to hypoxia after RIG-I removal (187). This observation implies that hypoxia may potentially participate in DM pathogenesis by regulating HIF-1α, stimulating RIG-I expression, and cooperating with other inflammatory factors. Furthermore, calcinosis is a common complication of chronic jDM, leading to long-term disability and sometimes death (183, 188). Clinical studies have reported that the use of JAK inhibitors (tofacitinib) has been shown to improve skin and muscle symptoms as well as calcinosis in DM patients (189). This may involve mitochondrial calcium storage and release imbalance mediated by the JAK/STAT3/HIF pathway, contributing to DM pathogenesis. Duvvuri et al. demonstrated that therapeutic targeting of mtROS effectively safeguards against mitochondrial damage in skeletal muscle cells, consequently mitigating the onset of calcinosis (190). Based on the above studies, we can speculate that regulating the hypoxic microenvironment and its associated HIF-1α upstream and downstream gene expression, such as VEGF, RIG-I and JAK/STAT3/HIF signaling pathways, may effectively reduce vascular inflammation and mitochondrial calcification in DM patients.

### 4.5 Sjögren’s syndrome

SS is a chronic autoimmune disorder characterized by dysfunction and inflammation of the lacrimal and salivary exocrine glands (191–193). Various factors such as hypoxia and infection induce activation of epithelial cells, leading to lymphocyte infiltration (especially CD4^+^ T cells) and release of inflammatory factors, thereby contributing to the pathogenesis of SS (191). It has been reported that in salivary gland tissues of SS patients and SS mouse models Th17 cells are highly increased, and HIF-1α can directly transcribe and activate RORγt to enhance Th17 development (97, 193), suggests that hypoxia may participates in the imbalance of T cell subsets and enhanced inflammation in SS. This is further supported by the finding of significantly increased genes involved in regulating cellular responses to hypoxic environments in primary SS patients’ minor salivary glands containing IL-21 (194). Moreover, HIF-1α appears to play a critical role in modulating salivary gland development and function under hypoxic conditions. Under hypoxic (5%O_2_) conditions, high expression of HIF-1α and VEGF was observed in mouse submandibular gland tissues, while HIF-1α mediated by BAY87-2243 inhibited salivary gland development (195). This suggests that hypoxia and HIF-1α activity may negatively impact salivary gland function in SS. Another study found that the C/T genotype (95% CI=0.09-0.52) and T allele (95% CI=0.12-0.58) of the HIF1A Pro582Ser polymorphism had a protective effect on primary SS (OR=0.22, *P*<0.01) and were susceptibility genetic factors for primary SS (192), further underscores the importance of HIF-1α activity in SS pathogenesis. In SS dry eye patients, upregulation of HIF-1α can activate the autophagy pathway, prevent damage to lacrimal acinar cells, and maintain normal lacrimal gland function (196). In primary SS renal tubulointerstitial injury, CD163-positive cells and high expression of HIF-1α were reported, where HIF-1α could promote macrophage polarization and participate in renal tubular injury, and early intervention to release HIF-1α could improve renal function in patients (197). Moreover, a Chinese herbal formula inhibits inflammatory cytokines (TNF-α, IL-6, and IL-1β) through IL-17, HIF-1α, and TNF-α signaling pathways, exerting anti-inflammatory effects in SS patients and providing a theoretical basis for its clinical application (198). In conclusion, hypoxic microenvironment and HIF-1α activity are closely associated with SS pathogenesis, affecting multiple organ systems and promoting disease progression. We speculate that modulating HIF-1α activity to improve hypoxia, exocrine gland function and relieve inflammation in SS patients may be a potential therapeutic option.

### 4.6 Ankylosing spondylitis

AS, as one type of AID, has a genetic susceptibility of over 90%, which is associated with human leukocyte antigen-B27 (HLA-B27) (199). The innate immune system plays a dominant role, with self-antibodies (such as anti-CD74 and anti-cyclic citrullinated peptide) as well as T cell activation and clonal expansion supporting its autoimmune components, and both autoinflammatory and autoimmune factors may continuously participate in its pathogenesis (199–202). Currently, the specific mechanism of low oxygen microenvironment in AS is still unclear, and there is limited research on hypoxia in AS. Ding et al. found that disruption of LncRNA-mediated competitive pathways may lead to the onset of AS. Among the 56 intact pathways identified in the AS group, with 35 of these encompassing competitive regulatory sub-pathways of LncRNA, the top three most prominent pathways were: 04010-1 (a subregion of the MAPK signaling pathway), 04062-1 (an important subregion of the chemokine signaling pathway), 04066-2 (part of the HIF-1 signaling pathway) (201). This indicates that LncRNA involving HIF may be involved in the pathogenesis of AS and may serve as a potential therapeutic target. Jiang J et al. found that the expression levels of the human hypoxia-associated genes, ANXA3 and SORL1, demonstrated a positively correlation with neutrophil counts (*P*<0.05). and the expression of neutrophil in AS patients was markedly elevated in comparison to the control group(*P*<0.01) (202). This finding suggests that these genes may play a role in modulating the neutrophil response under hypoxic conditions, thus involved in the pathogenesis of AS. In addition, increasing neutrophils may contribute to the progression of AS through phagocytosis, ROS production damaging tissue and impairing endothelial function, repairing damaged tissues, restricting NET production, or directly releasing NET to counter inflammation, which may be beneficial for the progression of AS (32, 61, 203). It is reasonable to speculate that the hypoxia microenvironment in AS lesions may serve as a trigger for immune cells, particularly neutrophils, to become activated and contribute to the pathogenesis of AS through the MAPK and HIF-1 signaling pathways. Therefore, improving the hypoxia microenvironment in AS lesions, perhaps through therapies aimed at enhancing oxygen delivery or reducing oxygen consumption, may help to dampen the inflammatory response and slow the progression of the disease. However, further research is needed to fully elucidate the mechanisms underlying the interactions between hypoxia, immune cells, and signaling pathways in AS pathogenesis.

In summary, hypoxic microenvironment participates in various the occurrence and development of various AIDs, including RA, SLE, MS, DM, SS, and AS (**Table 1**). Hypoxia conditions can affect the activity and function of immune cells, promote the release of inflammatory factors, and further aggravate the autoimmune reaction. It provides an unfavorable microenvironment for the occurrence and development of these complex diseases. Therefore, a thorough understanding of the mechanism of hypoxic microenvironment in AIDs is of great significance for the development of new therapeutic strategies.

**Table 1:** Hypoxic microenvironment participates in various AIDs.

Autoimmune Disease	Targeting HIF-1α to improve local hypoxic microenvironments		References
Rheumatoid arthritis	Proliferation, migration, and invasion of FLS	PI3K/AKT/HIF-1α	(131)
		NF-κB/HIF-1α	(132)
		STAT3/HIF-1α/fascin-1	(133)
		Notch-1	(134)
	Angiogenesis and inflammatory response	Notch-1	(134)
		CXCL12	(135)
		The downregulation of HIF-1α notably diminishes VEGF-mediated angiogenesis in FLS.	(136)
		PI3K/AKT/mTOR	(136)
	Joint erosion and bone degradation	Elevated expression of HIF-1α is associated with increased bone erosion in RA patients.	(137)
		HIF-1α promotes bone resorption mediated by OCs.	(138)
		ANGPTL4 compensates for HIF-1α’s inadequate stimulation of OC activity and promotes the proliferation and differentiation of OBs.	(138)
		HIF-1α induces the upregulation of RANKL in OBs, ultimately facilitating OCs-mediated bone resorption.	(137, 139)
		HIF-1α regulates Notch-1 to induce MMP2 and MMP9 expression in endothelial cells.	(132)
		The interaction between HIF-1α and NF-κB enhances the enzymatic activity of MMP2 and MMP9, leading to the disruption of tissue barriers and bone materials.	(130)
		IL-38 may upregulate osteogenic factors through the SIRT1/HIF-1α signaling pathway.	(140)
	Clinical application	The combination of DEX and ART can be used to treat RA by regulating the HIF-1α/NF-κB signaling pathway, controlling ROS clearance, and reversing macrophage polarization.	(75)
		Berberine inhibits the proliferation and adhesion of FLS in RA by regulating the MAPK/FOXO/HIF-1α signaling pathway.	(141)
		The injection of RBA into RA joints blocks the ERK/HIF-1α/GLUT1 signaling pathway, promotes phenotype transformation of macrophages, inhibits inflammatory cytokines and enhances tissue repair.	(142)
		Utilizing HIF-1α inhibitors, such as Roxadustat, prevents IEC death, maintains intestinal barrier function, and improves arthritis symptoms.	(143, 144)
Systemic Lupus Erythematosus	Erythrocyte maturation	HIF degradation damage during erythrocyte maturation leads to the accumulation of mitochondrial dense erythrocytes and subsequent mitochondrial dysfunction.	(150)
	T cell	CD4^+^ T cells play an important role in AIDs such as SLE through succinic acid accumulation, HIF-1α activation and mtROS production.	(154)
		Downregulation of mTOR in LN patients resulted in decreased STAT3 and Th17 cells in effector T cells.	(38, 156)
		Inhibition of AKT signaling pathway inhibits the migration of Th17 cells to the site of inflammation and effectively alleviates the chronic inflammatory process of LN.	(38, 156)
		mTOR activation triggers ROS production, and SIRT2 regulates the expression of IL-17A in these cells by regulating the mTORC1/HIF-1α/RORγt signaling pathway, thus participating in the pathogenesis of SLE.	(101, 155)
		mTORC1 may serve as an early diagnostic biomarker for SLE and its associated complications, including antiphospholipid syndrome.	(151)
		In LN mouse models, HIF-1α inhibitors (PX-478) delay the recruitment of T cells to the kidney, reverse the harmful effects of T cells on tissues, and ameliorate kidney injury.	(158)
	Macrophages	In renal macrophages, induction of glycolysis via the mTOR/HIF-1α signaling pathway inhibited immune complex-mediated inflammation and neutrophil recruitment.	(76)
		Luteolin inhibits HIF-1α expression and oxidative stress in macrophages and alleviates renal injury caused by LN infiltration by macrophages.	(157)
	Clinical application	In patients with photosensitive SLE, circulating miR-210 and HIF-1α levels were significantly increased and positively correlated.	(151, 152)
Multiple sclerosis	Tissue fibrosis	Hypoxia is an important factor in triggering fibrosis, inducing the deposition of ECM and increasing VEGF expression.	(162, 164, 165)
		Inhibition of the mtROS/HIF-2α signaling pathway may attenuate the deleterious role of CXCL4 in MS.	(166)
		Hypoxia promotes the autophagic activity of MS fibroblasts, and autophagy-mediated hypoxia-induced 2-methoxyestradiol inhibits MS collagen synthesis and EndoMT.	(167)
		Hypoxia may regulate EndoMT-related genes through TGF-β, NF-κB, TNF and mTOR signaling pathways, and participate in the pathogenesis of MS.	(171)
	Vascular pathology	In the early stage of MS, vascular endothelial injury and apoptosis up-regulate vasoactive substances and promote the migration of immune cells to the vasculature and peripheral tissues, leading to inflammation, oxidative stress and tissue hypoxia.	(162, 172, 173)
	Calcification	Vascular variation may predetermine MS patients’ susceptibility to osteolysis of the limb caused by finger ischemia and hypoxia, thereby promoting calcium plaque deposition in skin tissue.	(178)
		Hypoxia has been posited as a possible link between vascular dysfunction and calciphylaxis in MS.	(179)
		MS patients with and without calciphylaxis exhibited the higher levels of hypoxia-related proteins (GLUT1 and VEGF), with the highest levels observed in patients with calciphylaxis.	(182)
Dermatomyositis	Expression	In muscle fibers, endothelial cells express HIF-1α and HIF-1β, while muscle fibers express HIF-2α.	(185)
		HIF-1α was positive in juvenile DM muscle biopsy samples, and mRNA showed significantly increased HIF-1α expression.	(186)
		Double-positive macrophages of HIF-1α^+^ and CD 206^+^ were detected near capillaries (immunofluorescence staining).	(186)
		HIF-1α was present in DM muscle fibers and RIG-1 is a newly discovered HIF-induced gene containing HRE (Firefly/kidney test).	(187)
	Clinical application	Tofacitinib may improve skin and muscle symptoms and calcinosis in patients with DM through an imbalance in mitochondrial calcium storage and release mediated by the JAK/STAT3/HIF pathway.	(189)
		Targeting mtROS can prevent mitochondrial damage in skeletal muscle cells, thereby reducing calcinosis.	(190)
Sjögren’s syndrome	Salivary gland	Th17 cells were increased and HIF-1α directly transcribes and activates RORγt, a key transcription factor for Th17 development.	(97, 193)
		Genes that regulate cellular responses to hypoxic environments were increased in the small salivary glands of patients with primary SS containing IL-21.	(194)
		HIF-1α mediated by BAY87-2243 inhibited salivary gland development.	(195)
	Submandibular gland	In mouse models, high expression of HIF-1α and VEGF was observed under hypoxic conditions, and HIF-1α mediated by BAY87-2243 inhibited salivary gland development.	(195)
	Genetic predisposing factors	The HIF1A Pro582Ser polymorphism has also been implicated in SS susceptibility, with the C/T genotype and T allele conferring a protective effect against primary SS.	(192)
	Clinical application	HIF-1α has also been shown to promote macrophage polarization and participate in renal tubular injury in primary SS.	(197)
		In the context of dry eye, upregulation of HIF-1α in SS patients can activate the autophagy pathway.	(196)
		A Chinese medicine prescription has been shown to inhibit inflammatory factors in SS patients through IL-17, HIF-1α, and TNF-α signaling pathways.	(198)
Ankylosing spondylitis	Clinical application	LncRNA involving HIF may be involved in the pathogenesis of AS and may serve as potential therapeutic targets.	(201)
		Human hypoxia-related genes (ANXA3 and SORL1) may play a role in the regulation of neutrophil response and participate in the pathogenesis of AS.	(202)

In the treatment of RA, SLE, MS, DM, SS, AS, targeting HIF-1α to improve the local hypoxic microenvironment may involve research directions.

## 5 Therapeutic implications

### 5.1 Improving tissue oxygen supply

It was found that in an animal model of RA, the expression levels of ACPA, IL-17A, and HIF-1α in the group subjected to hyperbaric oxygen therapy (HBOT) were significantly reduced (*P*<0.05) compared to the non-HBO control group (204), This finding underscores the potential anti-inflammatory effects of HBO in RA, which may be mediated through increased tissue oxygenation, reduced inflammatory cell infiltration, and suppressed inflammatory factor production. the combined therapy of 2.5 mg/kg leflunomide and HBOT yielded significant improvements in foot edema and arthritis scores, compared to HBOT monotherapy. Additionally, this combination produced outcomes comparable to those achieved with 5 mg/kg leflunomide alone, particularly in terms of reduced inflammation (204). While leflunomide has been shown to be effective in treating RA, its use is accompanied by certain limitations and potential adverse effects, including diarrhea, nausea, alopecia, and hepatotoxicity, as reported in previous studies (205, 206). HBOT is effective in elevating the partial pressure of oxygen in the bloodstream and enhancing the dispersion of oxygen to tissues throughout the body, particularly to damaged joints, muscles, nerves, and the circulatory system. This increase in oxygen supply subsequently leads to improved local nutritional status and fosters tissue repair and regeneration. HBOT has garnered significant attention as a potential therapeutic intervention for arthritis, with support from several studies in the literature (207, 208). Collectively, these findings suggest that the integration of non-pharmacological strategies, such as HBOT, with pharmacological treatments may serve to optimize the therapeutic outcomes while potentially minimizing the dosage of medication required. We hypothesize that enhancing the oxygen content in the body through oxygenation or hyperbaric chamber therapy is expected to improve the oxygen supply to the tissues of patients with AIDs.

It has been extensively documented that HIF-1, functioning as a pivotal coregulator, exerts a positive regulatory influence on the expression of numerous pro-angiogenic genes and their corresponding receptors, including VEGF, placental growth factor (PIGF), and PDGF-B, among others. Consequently, this regulation promotes endothelial cell proliferation, migration, and lumen formation, which are crucial for angiogenesis. HIF-1 also modulates the expression of pro-angiogenic chemokines and their receptors, notably: stromal cell-derived factor-1α (SDF-1α) and sphingosine-1-phosphate (S1P), along with their cognate receptors such as CXCR4. This intricate regulation effectively orchestrates the migration of endothelial progenitors towards ischemic or injured regions, thereby actively contributing to the processes of vascular renewal, repair, and neovascularization. HIF-1 exerts a regulatory role over cell cycle proteins, particularly cyclins, and the Wnt signaling pathway, thereby fostering endothelial cell proliferation and division. This regulation serves as the cellular foundation necessary for the initiation and progression of angiogenesis (209). The intricate and multifaceted regulatory interplay between HIF and angiogenesis underscores the potential for novel therapeutic interventions targeting diseases characterized by aberrant angiogenesis. Jiang et al. found that the administration of the antirheumatic compound α-mangostin (MAN) in a rat model of adjuvant arthritis led to a marked suppression of elevated blood levels of TGF-β, IL-6, HIF-1α, and VEGF, accompanied by a reduction in the local expression of HIF-1α and VEGF in the joints. Furthermore, this treatment effectively mitigated the development of vascular opacities, as reported in their study (210). This suggests that the facilitation of neovascularization mediated by HIF signaling has the potential to enhance blood perfusion within injured tissues, thereby potentially mitigating the hypoxic environment experienced by AID patients.

### 5.2 Regulating hypoxia response pathways

#### 5.2.1 HIF-1α inhibitor

HIF-1α, as a core transcription factor for cells in response to hypoxic environment, is stabilized under hypoxic conditions and activates the expression of various genes. By regulating its activity, the inflammatory response induced by hypoxia can be significantly alleviated. Specifically, HIF-1α exerts a crucial influence on synovial inflammation, angiogenesis, and regulation of cellular metabolism in RA, among other processes. YC-1, an early identified HIF inhibitor, operates by diminishing the expression of HIF-1 target genes, including VEGF and inducible nitric oxide synthase (iNOS), through suppression of HIF-1α stability and transcriptional activity under hypoxic conditions, thereby modulating inflammatory responses and vascularization in RA (211). PX-12 has exhibited potent inhibition of HIF-1α accumulation and downstream gene expression across various tumor models, accompanied by a significant reduction in angiogenesis (212, 213). Furthermore, an array of HIF inhibitors, spanning benzopyran derivatives, sulfonamide derivatives, aromatic and heteroaromatic compounds, and steroidal HIF inhibitors, have demonstrated potent inhibitory effects on HIF in both *in vitro* and *in vivo* settings (214, 215).

However, the specific applications and exact effects of these inhibitors in AIDs need to be further explored in depth. In an antigen-induced arthritis (AIA) rat model, the administration of AMSP-30m, a HIF-1α inhibitor, was observed to elicit a marked reduction in foot swelling, arthritis index, and histopathological scores, alongside a notable decrease in the serum and synovial tissue levels of inflammatory cytokines, including IL-1β, IL-6, and TNF-α. Further analysis showed that AMSP-30m may exhibit strong anti-arthritic effects in AIA rats by promoting synovial cell apoptosis and reducing synovial angiogenesis (216, 217). Furthermore, naringenin, a promising bioactive compound, is postulated to modulate cellular adaptive responses to hypoxic conditions through its influence on HIF-1, TNF, and NF-κB signaling cascades, along with estrogen-mimetic activities. This modulation ultimately leads to a suppression of the production and secretion of inflammatory cytokines, including TNF-α, IL-1β, and IL-6, thereby regulating inflammatory reactions and mitigating tissue damage in T cell-mediated autoimmune diseases, such as RA, MS, and inflammatory bowel disease (218). Conversely, RBA-NPs, an innovative pH-responsive dual-target drug delivery system designed to target both CD44 and folate receptors, effectively downregulates macrophage glycolysis by inhibiting the ERK/HIF-1α/GLUT1 signaling axis. This, in turn, fosters the reprogramming of M1 into M2, thereby safeguarding immune homeostasis and averting excessive inflammatory reactions in RA (219). In summary, the study of HIF-1α and its inhibitors in the field of AID therapy not only provides an important basis for exploring new therapeutic strategies, but also reveals potential molecular targets, which heralds new directions and innovative breakthroughs in future therapeutic strategies.

#### 5.2.2 mTOR inhibitor

mTOR inhibitors exhibit a critical role in regulating immune responses and metabolic processes, effectively alleviating hypoxia-induced tissue damage. Specifically, rapamycin, a representative mTOR inhibitor, significantly inhibits IL-2-mediated T-cell proliferation by blocking the cell cycle transition from the G1 phase to the S phase, thereby attenuating excessive immune responses, especially in AIDs and transplant rejection (220–222). The binding of rapamycin to FK506 binding protein 12 (FKBP12) and its complex formation were found to be particularly effective in inhibiting mTOR, especially the mTORC1 isoform (223–225). Furthermore, mTOR inhibitors potently promote T-cell unresponsiveness, i.e., the diminished or loss of T-cell responsiveness to antigenic stimuli, in both *in vitro* and *in vivo* experiments (226, 227), which provides strong support for the reduction of rejection in contexts requiring immunosuppression, such as after organ transplantation. Additionally, mTOR is also involved in the regulation of immune-cell metabolic processes, in particular the Warburg effect that affects metabolic switching, which in turn regulates immune cell activity and function. In conclusion, mTOR inhibitors emerge as essential components within the realm of immunosuppression and metabolic modulation, underscoring their significance in these intricate biological processes.

In clinical application, sirolimus, an outstanding representative of mTOR inhibitors, is widely used to prevent rejection after solid organ transplantation and protect the safety of transplanted organs through its powerful immunosuppressive effect. For AIDs such as primary immune thrombocytopenia (ITP), sirolimus elicits suppressive immunomodulatory actions by augmenting the Treg cell proportion within the helper T lymphocyte subset, bolstering the count of naive T cells, triggering apoptosis in memory T cells, diminishing effector T cells, and enhancing megakaryocyte differentiation to expedite platelet production (228, 229). In addition, the use of temsirolimus alone in the treatment of advanced renal cell carcinoma was superior to IFN-α alone, with a significant prolongation of patient progression-free survival to 10.9 months (230). Another mTOR inhibitor, everolimus, has shown great potential in the treatment of various AIDs by precisely inhibiting the mTOR signaling pathway, regulating cell growth and proliferation, and thus influencing the immune response. Continuous treatment with everolimus for six years in patients with refractory SLE complicated by tuberous sclerosis has improved the activity status of LN, incrementally decreasing the corticosteroid dosage with minimal adverse effects (231), underscoring the long-term effectiveness and safety of everolimus in LN management. vistusertib markedly augmented its anti-meningioma activity through concurrent inhibition of both the mTORC1 and mTORC2 signaling pathways (232). In contrast, clinical trial results using apitolisib, an mTOR/PI3K inhibitor combination, in patients with metastatic renal cell carcinoma showed shorter progression-free survival and a higher incidence of grade 3-4 toxicity (233). However, along with improved efficacy, close monitoring of toxicity is needed to ensure the safety of treatment.

Studies have demonstrated that celastrol inhibits the PI3K/AKT/mTOR signaling pathway, increases the autophagosome level of FLS, decreases the phosphorylation of mTOR and AKT, and induces autophagy, thus ameliorating RA (234). Baicalein has been shown to mitigate RA by inhibiting the proliferation of FLS and EndoMT, while inducing apoptosis through the inhibition of the PI3K/Akt/mTOR signaling axis (235). Artesunate exerts effects on chondrocyte proliferation and apoptosis through the PI3K/AKT/mTOR signaling pathway in a rat model of RA (236). Phlorizin has been found to reduces autophagy through modulation of the Akt/PI3K/mTOR pathway, which protects against inflammation and reduces synovial tissue damage in RA (237). Collectively, these mTOR inhibitors demonstrate potent pharmacological activities and exhibit promising prospects for therapeutic interventions aimed at mitigating RA. Taken together, these findings underscore the therapeutic potential of mTOR inhibitors in diverse conditions, including cancer, immunosuppression post-organ transplantation, and AIDs, through their ability to modulate critical upstream and downstream signaling molecules.

### 5.3 Antioxidant therapy

Under the hypoxic environment, the rational application of antioxidants, including vitamin C, vitamin E, and N-acetylcysteine (NAC), with meticulous consideration of optimal dosage, combination, and timing of administration, exhibited remarkable antioxidant synergism, effectively mitigating cellular damage inflicted by free radicals, thereby preserving cellular integrity and health (238). The combined application of vitamin C and vitamin E forms a complementary mechanism, whereby vitamin C promotes the regeneration of oxidized vitamin E to build an efficient antioxidant cycle. Furthermore, the antioxidant potential can be augmented through the incorporation of NAC, serving as a precursor for glutathione (GSH), thereby elevating intracellular GSH levels. GSH, a pivotal non-enzymatic antioxidant, plays a fundamental role in the elimination of free radicals and the preservation of redox balance. Research has demonstrated that the combination of vitamin E/C and NAC, even at low doses *in vitro*, effectively diminished the generation of NET while notably augmenting the total antioxidant capacity (TAC) and the GSH/oxidized glutathione disulfide (GSSG) ratio. Additionally, it reduced ROS levels and exhibited robust antioxidant protective effects in neutrophil models (238, 239). Apart from the aforementioned antioxidants, naringenin, another potent antioxidant, has exhibited the capability to scavenge free radicals and mitigate oxidative stress, which is important for the protection of cells from oxidative damage induced by autoimmune responses (218). In the treatment of inflammatory diseases such as RA, the combination of ART and DEX not only plays a role by inhibiting the HIF-1α/NF-κB signaling pathway, but also promotes ROS scavenging and macrophage polarization from M1 to M2, which modulates inflammatory responses at multiple levels, providing a new perspective for antioxidant treatment strategies (75).

It was found that artemisinin and its derivatives counteracted oxidative stress in RA. This was achieved by up-regulating NF-E2-related factor-2 (NRF-2), kelch-like ECH-associated protein 1 (KEAP-1), and heme oxygenase 1 (HO-1), which are essential components of the antioxidant response pathway. Furthermore, HIF-1α downregulation was observed, concurrent with decreased expression of MMP-2 and MMP-9, which contribute to inflammation (240). Consequently, these changes led to a marked enhancement of *in vivo* antioxidant defense mechanisms. Another independent study showed that extra virgin olive oil (EVOO) in a mouse model of SLE significantly up-regulated the expression of NRF-2 and HO-1 proteins and effectively ameliorated the activation status of JAK/STAT, MAPK, and NF-κB pathways (241). These results imply that EVOO possesses a potential prophylactic effect on immune-mediated inflammatory disorders, notably SLE, via bolstering antioxidant capabilities, thereby reinforcing the crucial role of augmenting antioxidant defenses in mitigating the severity of these conditions.

### 5.4 Immunomodulatory therapy

#### 5.4.1 Immunosuppressants

Immunosuppressants utilized in the therapy of AIDs can be classified into two distinct categories: selective and non-selective, depending on their underlying mechanisms of action. Selective immunosuppressants, encompassing selective T-cell inhibitors like cyclosporin A, tacrolimus, and fingolimod, as well as rituximab and abatacept, precisely target specific immune cells or molecules, exerting their effects with precision; whereas nonselective immunosuppressants, which comprise glucocorticoids, cyclophosphamide, antimetabolites such as methotrexate and leflunomide, as well as herbal extracts like tripterygium wilfordii and sabia japonica maxim, exhibit a broader spectrum of effects, impacting multiple aspects of the immune system (242, 243) Although immunosuppressants are effective in reducing the overreaction of the immune system to pathological environments such as hypoxia, the accompanying heightened risk of complications, such as the development of malignant tumors, infectious diseases, and osteoporosis, underscores the need for an ideal immunosuppressant agent that is not only highly selective and fast-acting but also efficacious, with the capability to reverse its effects upon drug discontinuation, and possesses minimal side effects without compromising normal immune function. Unfortunately, to date, no single drug has been able to fully meet all these stringent criteria (222, 240, 244).

Recent studies have demonstrated that artemisinin-type drugs effectively impede receptor-mediated signaling cascades by inhibiting an array of signaling pathways, including IL-1, TNF-α, β3-integrin, RANKL, Toll-like receptor, and growth factor receptor pathways, and by modulating crucial signaling molecules such as JNK, PI3K, AKT, ERK, and MAPK, among others. Artemisinin markedly diminishes the inflammatory response, primarily through downregulation of NF-κB activity and its downstream cytokines, chemokines, and gene expressions, while also extending its regulatory effects to encompass transcription factors such as mTOR, activator protein-1 (AP1), HIF-1α, STAT, and NRF-2, thereby enhancing its anti-inflammatory and immunomodulatory capabilities (240). *In vivo* experiments have demonstrated the pronounced therapeutic efficacy of artemisinin-type drugs in treating rheumatic diseases, including RA, osteoarthritis, lupus erythematosus, arthrosis, gout and other inflammatory conditions and AIDs. These drugs have been shown to reduce the proliferation of fibrous connective tissue, chondrocytes, and capillaries in RA patients (245) and correct immune imbalances and abnormal signal transduction in patients with SLE (246). Furthermore, the combination of artemisinin with other therapeutic agents, such as immunomodulators, chemotherapeutic agents, and radiotherapy, exhibits promising synergistic effects and the potential to mitigate side effects. However, caution must be exercised to monitor for potential toxic reactions, including neurotoxicity and hematotoxicity, thus necessitating rigorous evaluation of such combinations. For instance, the combination of artesunate with immunomodulators, including methotrexate, tretinoin, and azathioprine, elicited superior macrophage apoptosis compared to the individual administration of any of these drugs (247). There may also be an antagonistic effect in terms of toxicity, i.e., a reduction in the side effects of the other drugs (248, 249). In conclusion, artemisinin analogs demonstrate significant potential in the immunosuppressive management of AIDs, and their distinctive multi-target and multi-mechanistic mode of action presents a promising avenue for the development of innovative immunosuppressive agents that exhibit both high efficacy and low toxicity.

#### 5.4.2 Biologics

Anti-TNF antibodies and IL-6 inhibitors specifically modulate immune system function, thereby effectively reducing the inflammatory response. Tocilizumab, a humanized anti-IL-6 receptor monoclonal antibody, has demonstrated rapid and long-lasting efficacy by directly targeting the causative agents of RA, and is particularly indicated for adults with moderately-to-severely active RA who have had an inadequate response to conventional antirheumatic drugs (250). Additionally, tolizumab has demonstrated therapeutic efficacy in managing systemic juvenile idiopathic arthritis, adult Still’s disease, and Crohn’s disease (251). A TNF-α, a pivotal pro-inflammatory cytokine, exhibits a strong association with numerous cytokines, including IL-1, IL-6, IL-8, and VEGF, and therefore inhibitors of TNF-α occupy an important role in immunosuppressive therapy (252–254). TNF-α modulates the expression of MMP-9 via diverse molecular pathways, fostering the degradation of the ECM. This, in turn, influences tumor migration and indirectly augments the release of tumor growth-promoting factors (255).The FDA has approved a variety of anti-TNF-α biologics, such as infliximab and etanercept, for the treatment of moderate to severe RA, and the etanercept has been extended to juvenile RA, and the indication for etanercept has been extended to the treatment of juvenile RA (256). Adalimumab, a fully humanized monoclonal antibody specific to TNF-α, is primarily utilized in the treatment of moderate to severe RA, psoriatic arthritis, AS, and Crohn’s disease. It effectively mitigates inflammation and bone degradation by specifically inhibiting TNF-α (137, 253, 257). Thalidomide, as a drug capable of inhibiting TNF-α protein synthesis, effectively slows down the progression of many cancers, including multiple myeloma, by blocking the synthesis of several growth factors, including VEGF, basic fibroblast growth factor, and hepatocyte growth factor, as well as inhibiting the synthesis of tumor deoxyribonucleic acid (DNA) (258, 259). However, studies have shown that low concentrations of TNF-α actually have a pro-angiogenic effect, which is necessary for certain physiological and pathological processes. However, studies have shown that TNF-α, at low concentrations, potently elicits a pro-angiogenic effect, whereas its role reverses to an anti-angiogenic function at higher concentrations (260). The dichotomous roles exhibited by TNF-α underscore the intricate interplay that exists between angiogenesis and inflammation. In addition, the use of TNF-α inhibitors is accompanied by certain risks. Studies have shown that RA patients treated with TNF-α inhibitors have an increased risk of developing leukemia and lymphoma (261). Another study also noted an increased risk of lymphoma in RA patients treated with etanercept or infliximab (258). It was also found that there was also a significant dose-dependent relationship between the dose of TNF-α inhibitors used and the risk of malignancy (262). Consequently, when employing drugs that modulate TNF-α in therapeutic interventions, it is imperative to meticulously assess the risks and benefits for patients, thereby ensuring the safety and effectiveness of the treatment.

### 5.5 Metabolic regulation therapy

Metabolic modulation therapy has demonstrated potential therapeutic value for a variety of diseases, including RA, by regulating cellular metabolic pathways, improving cellular energy metabolism under hypoxic conditions and reducing metabolic stress responses. Among them, metabolic modulators, exemplified by metformin, effectively regulate the Treg/Th17 balance of patients with RA through the activation of AMPK and the inhibition of key signaling pathways, such as mTOR, STAT3, and HIF-1, attenuating the inflammatory response and inhibiting osteoclast formation, thereby alleviating disease progression (219). Compounds like dimethyl malonate (MAN) facilitate the recovery of energy metabolism by mitigating oxidative stress, optimizing glucose utilization, minimizing the accumulation of glycolytic byproducts, and reinstating the functionality of LDH, thereby enhancing cellular metabolic efficiency (210). These strategies improve the metabolic environment of cells while enhancing the overall defense of the body. Furthermore, the supplementation of energy substrates, including glucose and lactate, significantly augments the energy supply of cells under hypoxic conditions, thereby ensuring the sustained maintenance of normal cellular functions and metabolic homeostasis. Glucose metabolism and ATP production are involved in immune cell activation, proliferation and signal transduction as well as transport and effector functions, thus contributing to immune response programming and helping the host to adapt to changes in the microenvironment (263), therefore, glucose supplementation emerges as an efficacious strategy for bolstering cellular energy supply and fostering optimal cellular function, underscoring its therapeutic potential. A class of probiotics has been found to mimic endogenous immune metabolic regulation in dendritic cells by generating lactic acid and activating the HIF-1α/NADH dehydrogenase (ubiquinone)-1α subcomplex 4-like 2 (NDUFA4L2) signaling pathway, which reduces mtROS production and thus inhibits auto immune T cell overactivation (264), opening new avenues for the treatment of AIDs, including EAE. These findings underscore the important role of metabolic modulation therapies in fostering immune homeostasis and facilitating the restoration of organismal health.

In summary, these therapeutic implications of drugs on AIDs (**Table 2**), are designed to optimize the hypoxic microenvironment through multifaceted pathways, ultimately curbing inflammation and mitigating tissue damage, thereby achieving the desired therapeutic outcomes for autoimmune diseases.

**Table 2:** Therapeutic implications of drugs on AIDs.

Mechanism		Medication	Diseases	Possible therapeutic implications	Reference
Improving tissue oxygen supply	Oxygen therapy	Leflunomide and hyperbaric oxygen therapy	RA	Increase tissue oxygen supply, decrease inflammatory cell infiltration, and inhibit production of inflammatory factors	(204)
	Angiogenesis therapy	α-mangostin	Osteoarthritis	Limit the rise of blood TGF-β, IL-6, HIF-1α and VEGF levels, reduce the expression of HIF-1α and VEGF locally in the joints, and curb the generation of vascular opacities	(210)
Regulating hypoxia response pathways	HIF-1α Inhibitors	YC-1	RA	Reduce VEGF and iNOS expression and modulates inflammatory response and angiogenesis	(211)
		PX-12	Tumors	Inhibit HIF-1α accumulation and its downstream gene expression and reduce angiogenesis	(212, 213)
		AMSP-30m	AIA rat	Reduce the production of inflammatory factors in serum and synovial tissue, promote synovial cell apoptosis and reduce synovial angiogenesis	(216, 217)
		Naringenin	RA, MS and inflammatory bowel disease	Affect HIF-1, TNF, NF-κB signaling pathways and estrogen-like effects; reduce the production and release of inflammatory factors such as TNF-α, IL-1β and IL-6; regulate T cells	(218)
		RBA-NPs	RA	Blockade of the ERK/HIF-1α/GLUT1 signaling pathway to downregulate glycolysis levels in macrophages promotes reprogramming of the M1 to M2 transition	(219)
	mTOR Inhibitors	Sirolimus	Rejection after organ transplantation, ITP	Elevate the proportion of Treg cells in the helper T lymphocyte subset	(228, 229)
		Temsirolimus	Advanced renal cell carcinoma		(230)
		Everolimus	Patients with refractory SLE complicated by tuberous sclerosis, LN	Inhibition of the mTOR signaling pathway regulates cell growth and proliferation, which in turn influences the immune response	(231)
		Vistusertib	Meningioma	Inhibition of mTORC1 and mTORC2 pathways	(232)
		Apitolisib	Metastatic renal cell carcinoma	mTOR/PI3K inhibitors	(233)
Antioxidant therapy	Antioxidants	Vitamin C, Vitamin E and NAC		Reduces NET formation; reduces ROS	(238, 239)
		ART and DEX	Inflammatory diseases such as RA	Inhibition of the HIF-1α/NF-κB signaling pathway; promotion of ROS clearance and macrophage polarization from M1 to M2 phenotype	(75)
	Activating Endogenous Antioxidant Systems	Artemisinin and its derivatives	RA	Up-regulation of NRF-2, KEAP - 1 and HO-1 and down-regulation of HIF-1α to counteract oxidative stress	(240)
Immunomodulatory therapy	Immunosuppressants	Artemisinin-type drugs	RA, osteoarthritis, lupus erythematosus, arthrosis, gout and other inflammatory conditions and AIDs	Inhibition of NF-κB regulates immune cells and cytokines to correct immune imbalance; activation of NRF-2/HO-1 pathway reduces inflammatory oxidative stress.	(240)
	Biologics	Tocilizumab	RA, systemic juvenile idiopathic arthritis, adult Still’s disease, and Crohn’s disease	Anti-IL-6 receptor	(250)
		Infliximab, Etanercept	RA	Anti-TNF-α	(256)
		Adalimumab	RA, psoriatic arthritis, AS, and Crohn’s disease	Specific blockade of TNF-α to attenuate inflammation and bone destruction	(137, 253, 257)
		Thalidomide	cancers	Inhibition of TNF-α protein synthesis; inhibition of tumor DNA synthesis	(258, 259)
Metabolic regulation therapy	Metabolic Regulators	Metformin	RA	Activates AMPK and inhibits key signaling pathways such as mTOR, STAT3 and HIF-1 to regulate Treg/Th17	(219)
		Dimethyl malonate	AIDs	Relieves oxidative stress and optimizes glucose metabolism	(210)
	Supplementing Energy Substrates	Glucose	AIDs	Produces ATP; involved in the activation of immune cells	(263)
		Probiotics	EAE	Generation of lactate and activation of the HIF-1α/NDUFA4L2 signaling pathway reduces mtROS production and inhibits T-cell-driven autoimmune responses	(264)

These strategic approaches are designed to optimize the hypoxic microenvironment through multifaceted pathways, ultimately curbing inflammation and mitigating tissue damage, thereby achieving the desired therapeutic outcomes for autoimmune diseases.

## 6 Discussion

An increasing body of research evidence indicates a close association between hypoxic microenvironments and the occurrence and development of various AIDs, including RA, SLE, MS, DM, SS, and AS. For instance, HIF-1α is implicated in the proliferation, migration, and invasion of FLS in RA patients, regulating angiogenesis and inflammatory responses (136), as well as joint erosion and bone degradation (137). Downregulation of HIF-1α via the mTOR1 pathway may mitigate the Warburg effect in SLE patients, hindering T-cell differentiation, inhibiting glycolytic metabolism, and thereby modulating inflammatory and immune responses (101, 155). Additionally, HIF-1 modulates the pathogenesis and pathological processes of MS patients by regulating the hypoxic microenvironment, fibrosis, vascular dysfunction, immune inflammation, and skin calcification reactions (165, 173, 179). Modulation of HIF-1 and its upstream and downstream genes may contribute to the pathological processes of angiogenesis, inflammation, and complications such as calcinosis in DM patients (185, 189). Adjusting HIF-1α activity can influence Th17 development, inflammatory cytokine release, and exocrine gland function in SS patients, thereby impacting SS progression (97, 198). Furthermore, the HIF-1 signaling pathway interacts with other immune and inflammatory-related signaling pathways (MAPK, chemokine signaling pathways), collectively influencing the pathological process of AS (201).

Under hypoxic microenvironments, the activation of the mTOR signaling pathway by HIF-1α may contribute to the amplification of the Warburg effect, resulting in the upregulation of genes involved in glycolysis. This, in turn, facilitates enhanced glucose uptake and utilization by cells, accompanied by a concurrent increase in lactate production (50–52). This metabolic reprogramming serves as a crucial source of energy for rapidly proliferating cells, notably tumor cells and inflammatory cells, augmenting their capacity to adapt to hypoxic conditions. Consequently, it may potentially exacerbate autoimmune pathological processes that are intimately linked to hypoxia (50, 56). Furthermore, HIF-1α regulates the expression of proangiogenic factors (VEGF, PIGF and PDGF-B), proangiogenic chemokines (SDF-1α and S1P), and cyclins, thereby orchestrating the orderly participation of endothelial cells in angiogenesis (209). In AIDs, HIF-1α-mediated angiogenesis may augment blood perfusion within damaged tissues, ameliorating local hypoxia, yet potentially fostering the infiltration of inflammatory cells and exacerbating autoimmune responses (210). Moreover, HIF-1α interacts with TCR, activating HIF-responsive genes, influencing T-cell differentiation and function, and modulating Th1, Th2, Th17, and Treg subsets, thereby regulating inflammatory responses, maintaining immune homeostasis, and suppressing autoimmune reactions (90–92). In AIDs, downregulation of HIF-1α significantly diminishes the number of IL-10-secreting B cells, exacerbating collagen-induced arthritis and EAE (89). Additionally, HIF-1α modulates the activation and function of immune cells, including macrophages, dendritic cells, and MDSCs, thereby influencing the production and release of inflammatory factors. These findings indicate that in the hypoxic microenvironment, the activation of the key regulator HIF-1α significantly promotes the progression of AIDs, evident in metabolic reprogramming, angiogenesis, and immune cell activation, thereby exacerbating inflammatory responses and autoimmune pathological processes.

Currently, therapeutic strategies for AIDs primarily encompass immunosuppressants, biologics, and glucocorticoids among other pharmaceutical classes. The mechanism of these drugs lies in inhibiting excessive immune system activation, subsequently leading to the effective alleviation of inflammatory responses and tissue damage. Nevertheless, these therapies are inevitably accompanied by extensive immunosuppressive effects, potentially precipitating a range of adverse reactions, including but not limited to increased infection risk and the potential development of secondary malignancies (265–269). For instance, NSAIDs used by RA patients to inhibit prostaglandin physiological functions can elicit gastrointestinal reactions like gastric and duodenal ulcers, bleeding, and cardiovascular and hematological disturbances stemming from coagulation disturbances. Similarly, biological agents may predispose patients to infections and further immunosuppression (270). The advantages of targeting hypoxia and HIF-1α pathways for AID treatment are multifaceted. Firstly, precision: potential therapeutic strategies targeting hypoxia and HIF-1α can precisely address the pathophysiological processes of hypoxia and inflammation, minimizing unintended impacts on normal immune function. Secondly, multi-pathway modulation: by improving tissue oxygenation, modulating hypoxic response pathways, antioxidant therapy, immune modulation, and metabolic regulation, these approaches mitigate hypoxic tissue damage and inflammation, thereby inhibiting inflammatory reactions and metabolic alterations, ultimately alleviating the autoimmune pathological progression in AID patients. And finally, potential synergy: therapies targeting hypoxia and HIF-1α may have the potential to create synergies with existing drugs compared to existing treatments, thereby enhancing the therapeutic effect (233). It is also possible to reduce the dose and frequency of monotherapy, thereby reducing the incidence of side effects. For those patients who do not respond well to traditional therapies, the combination of hypoxic targeted therapy and traditional therapy offers a new option.

Our hypothesis suggests that future research endeavors will further elucidate the stabilization mechanisms of HIF-1α under hypoxic conditions and its precise regulatory interactions with downstream genes, identifying the specific structural domains involved in HIF-1α’s binding to diverse target genes, thereby providing a theoretical foundation for the design and synthesis of small-molecule HIF-1α inhibitors with enhanced specificity and affinity. Secondly, we aim to explore the roles of other hypoxia-related factors in the progression of AIDs, this includes a thorough analysis of the stability and activation mechanisms of HIF-2α under hypoxic conditions, and investigating the interactions and differences between HIF-2α and HIF-1α in the progression of AIDs, to optimize therapeutic outcomes and minimize side effects through the combined administration of HIF-1α and HIF-2α inhibitors (27, 166). Furthermore, personalized medicine approaches can be formulated based on patients’ responses to hypoxic environments and the expression levels of molecules such as HIF-1α. Conducting genetic sequencing and bioinformatic analysis for AID patients can identify genetic variations associated with hypoxic response and HIFs signaling pathways, enabling the identification of patient subgroups with specific genetic profiles that may exhibit increased sensitivity or resilience to hypoxia-targeted therapies. As gene sequencing and bioinformatic technologies continue to advance, personalized treatment approaches are poised to become viable options in AID management. Therefore, the development of more specific HIF-1α and HIF-2α inhibitors, alongside the exploration of personalized medicine, holds promise for bringing about potential solutions to improve treatment outcomes for AID patients.

The hypoxic microenvironment holds profound clinical implications in AIDs, potentially unveiling novel diagnostic biomarkers and informing management strategies tailored to patients across various disease stages (271–273). HIF-1α and its downstream effectors, such as VEGF and PDK1, alongside markers of metabolic reprogramming, including LDH and pyruvate kinase, play crucial roles in inflammation, angiogenesis, and metabolic modulation, intimately intertwined with the pathological mechanisms underlying AIDs (35, 210). The influence of low oxygen and HIF-1α may vary across the stages of AIDs, facilitating the assessment of disease severity and progression by monitoring alterations in these biomarkers. For example, early disease stages may necessitate aggressive anti-inflammatory and immunomodulatory interventions, whereas intensified vascular protection and metabolic modulation strategies may be required during disease progression (178–180). By simulating the hypoxic microenvironment or regulating the activity of molecules such as HIF-1α, a series of adaptive changes are induced in immune cells, thereby improving disease symptoms and improving patients’ quality of life. However, the specific effects and safety of hypoxic therapy still need to be verified by further clinical trials.

Despite the profound insights gained from studying the relationship between hypoxic microenvironments and AIDs, translating these findings into clinical practice faces numerous challenges and limitations. Firstly, the heterogeneity of low oxygen levels across different tissues adds complexity to understanding and applying these discoveries, necessitating tissue-specific investigations. For instance, the expression and function of HIF-1α in bone, kidney, and brain vary significantly, underscoring the need for targeted research (137, 157). Secondly, local hypoxic environments are influenced by multifarious factors, including blood flow velocity, inflammation intensity, and tissue metabolic status, complicating the accurate assessment of hypoxia levels in clinical settings. Furthermore, low oxygen exerts its effects through intricate signaling pathways, including HIF-1, NF-κB, and non-hypoxia-stimulated regulatory pathways, which interact in complex ways, adding to the complexity of understanding and regulating these processes (**Figure 2B**). Consequently, targeted therapies aimed at hypoxic signaling pathways necessitate precise control of drug dosages and timing to mitigate side effects and the development of resistance.

Hence, future endeavors must aim to overcome these challenges by delving deeper into the mechanisms of hypoxia, developing novel diagnostic biomarkers and therapeutic strategies, and integrating advanced technologies with refined treatment protocols to enhance care for AID patients. Leveraging advanced technologies such as high-throughput sequencing, single-cell sequencing, and proteomics can provide a more comprehensive understanding of the mechanisms of hypoxia across tissues and cells, underpinning precision medicine. Personalized treatment plans can be devised by integrating patients’ genetic profiles, disease stages, and clinical manifestations. For instance, genetic testing can determine a patient’s sensitivity to hypoxia-related drugs, facilitating the optimization of treatment dosages and regimens. Multidisciplinary collaboration should be used to comprehensively evaluate the condition of AID patients and formulate comprehensive treatment plans, so as to improve the treatment effect.

In conclusion, the clinical significance of hypoxia in AIDs is undeniable, yet it faces multiple challenges and constraints. This study presents a theoretical framework elucidating the pivotal role of the core molecule HIF-1α in AID pathogenesis within hypoxic microenvironments, offering vital insights for the development and potential application of targeted therapies aimed at this pathway.

## Glossary

AIDs: autoimmune diseases
RA: rheumatoid arthritis
SLE: systemic lupus erythematosus
MS: multiple sclerosis
DM: dermatomyositis
HIF: hypoxia-inducible factor
PGK: phosphoglycerate kinase
LDHA: lactate dehydrogenase A
pVHL: the tumor suppressor protein Von Hippel-Lindau Tumor Suppressor Protein
VEGF: vascular endothelial growth factor
ODD: oxygen-dependent degradation domain
PHDs: prolyl hydroxylases
Ub: ubiquitin proteins
FIH: factor-inhibiting HIF
TAD: transactivation domain
HREs: hypoxic response elements
GLUT1: glucose transporter 1
EPO: erythropoietin
MAPK: mitogen-activation protein kinase
ERK: extracellular protein kinases
EGF: epidermal growth factor
IGF-1: insulin-like growth factor-1;PI3K, phosphoinositide 3-kinase
PKB/AKT: protein kinase B
mTOR: the mechanistic target of rapamycin kinase
STAT3: signal transducer and activator of transcription 3
AMPK: AMP-activated protein kinase
JAK: Janus tyrosine kinase
NLRP3: nucleotide-binding oligomerization structure-like receptor family pyrin domain protein 3
Treg: regulatory T cells
TNF-α: tumor necrosis factor α
TAK1: transforming growth factor-activated kinase 1
HMGB1: hypoxia-induced high mobility group proteins 1
JNK: c-Jun N-terminal kinase
SIRT: silence information regulator
Ang1: angiopoietins1
PDGF-BB: platelet-derived growth factor-BB
IL-1β: interleukin-1β
ROS: reactive oxygen species
NF-κB: nuclear factor kappa-B
GRK2: G protein-coupled receptor kinase 2
PDK1: phosphoinositide-dependent protein kinase-1
ATP: adenosine triphosphate
NETs: neutrophil extracellular traps
GM-CSF: granulocyte-macrophage colony stimulating factor
BLyS: B-lymphocyte stimulator
DMARDs: disease-modifying anti-rheumatic drugs
LPS: lipopolysaccharide
SS: Sjögren’s syndrome
LncRNA Dpf3: long non-coding ribonucleic acid Dpf3
microRNA: micro-ribonucleic acid
RORγt: RAR-related orphan receptor gamma t
MDSCs: myeloid-derived suppressor cells
SPNTi: sono-activatable semiconducting polymer nanopartners
SDT: sonodynamic therapy
TPZ: tirapazamine
ICD: immunogenic cell death
MLS: macrophage-like synoviocytes
FLS: fibroblast-like synoviocytes
CIA: collagen-induced arthritis model
OA: osteoarthritis
EndoMT: endothelial-to-mesenchymal transition
CXCL12: chemokine (C-X-C motif) ligand 12
OCs: osteoclasts
ANGPTL4: angiopoietin-like 4
OBs: osteoblasts
RANKL: receptor activator of NF-κB ligand
MMP2: matrix metalloproteinase 2
DEX: Dexamethasone
ART: artesunate
RBA: roburic acid
RIPK3: receptor-interacting protein kinase 3
IECs: intestinal epithelial cells
LN: lupus nephritis
PLN: proliferative lupus nephritis
mtROS: mitochondrial ROS
RET: reverse electron transfer
mTORC1: mTOR complex 1
MRL/lpr mice: metabolic reprogramming in lupus-prone mice
ECM: extracellular matrix
TGF-β: transforming growth factor-β
jDM: juvenile DM
RIG-I: retinoic acid-inducible gene I
HLA-B27: human leukocyte antigen-B27
HBOT: hyperbaric oxygen therapy
PIGF: placental growth factor
SDF-1α: stromal cell-derived factor-1α
S1P: sphingosine-1-phosphate
MAN: α-mangostin
iNOS: inducible nitric oxide synthase
AIA: antigen-induced arthritis
FKBP12: FK506 binding protein 12
ITP: immune thrombocytopenia
NAC: N-acetylcysteine
GSH: glutathione
GSSG: oxidized glutathione disulfide
TAC: the total antioxidant capacity
NRF-2: NF-E2-related factor-2
KEAP-1: kelch-like ECH-associated protein 1
HO-1: heme oxygenase 1
EVOO: extra virgin olive oil
AP1: activator protein-1
DNA: deoxyribonucleic acid
LDH: lactate dehydrogenase
NDUFA4L2: NADH dehydrogenase (ubiquinone)-1α
EAE: experimental autoimmune encephalomyelitis.
